# A Study of the Influence of Sex on Genome Wide Methylation

**DOI:** 10.1371/journal.pone.0010028

**Published:** 2010-04-06

**Authors:** Jingyu Liu, Marilee Morgan, Kent Hutchison, Vince D. Calhoun

**Affiliations:** 1 The Mind Research Network, Albuquerque, New Mexico, United States of America; 2 Department of Electrical and Computer Engineering, University of New Mexico, Albuquerque, New Mexico, United States of America; 3 Department of Psychology, University of New Mexico, Albuquerque, New Mexico, United States of America; Baylor College of Medicine, United States of America

## Abstract

Sex differences in methylation status have been observed in specific gene-disease studies and healthy methylation variation studies, but little work has been done to study the impact of sex on methylation at the genome wide locus-to-locus level or to determine methods for accounting for sex in genomic association studies. In this study we investigate the genomic sex effect on saliva DNA methylation of 197 subjects (54 females) using 20,493 CpG sites. Three methods, two-sample T-test, principle component analysis and independent component analysis, all successfully identify sex influences. The results show that sex not only influences the methylation of genes in the X chromosome but also in autosomes. 580 autosomal sites show strong differences between males and females. They are found to be highly involved in eight functional groups, including DNA transcription, RNA splicing, membrane, etc. Equally important is that we identify some methylation sites associated with not only sex, but also other phenotypes (age, smoking and drinking level, and cancer). Verification was done through an independent blood cell DNA methylation data (1298 CpG sites from a cancer panel array). The same genomic site-specific influence pattern and potential confounding effects with cancer were observed. The overlapping rate of identified sex affected genes between saliva and blood cell is 81% for X chromosome, and 8% for autosomes. Therefore, correction for sex is necessary. We propose a simple correction method based on independent component analysis, which is a data driven method and accommodates sample differences. Comparison before and after the correction suggests that the method is able to effectively remove the potentially confounding effects of sex, and leave other phenotypes untouched. As such, our method is able to disentangle the sex influence on a genome wide level, and paves the way to achieve more accurate association analyses in genome wide methylation studies.

## Introduction

DNA methylation occurs on the C5 position (the 5^th^ carbon of the cytosine pyrimidine ring) of CpG dinucleotides along the DNA chain, and forms one of the epigenetic mechanisms controlling and modulating gene expression. It is not only essential for normal cellular development, but also may be associated with formation of diseases. Cancer, for example, has been related to genome-wide hypomethylation coinciding with gene specific hypermethylation [1]. A few new studies also show that methylation change of specific genes is associated with mental illness, such as hypomethylation of *MB-COMT* in schizophrenic and bipolar disorder patients [2], [3], [4], [5].

In addition to disease, inter-individual differences in healthy subjects are also observed and are influenced by factors like age, sex and tissue type [6]. Sex differences have been discussed with contradictory results. Eckhardt et al. [7] studied 2,524 loci on chromosome 6, 20 and 22 in 12 different tissues in 43 samples, and could not find any statistical difference between male and female samples. In contrast, Sarter et al. reported significant sex differences in four autosomal genes, and suggested that sex is at least as strong a predictor of methylation in certain genes as age [8]. Moreover, conflicting results have been drawn regarding whether females or males have higher methylation levels. A tendency toward higher methylation levels in males was identified in three regions at *PEG3*, *NESP55 (GNAS)* and *H19* imprinted genes and two additional loci at *Xq28* (*F8* gene) and at 19q13.4 [9]. Expression level of *DNMT3b* (a DNA methyltransferase) in human liver is significantly higher in females than males, which potentially influences DNA methylation status [10]. In methylation-disease studies, females are shown to be 8.8 time more likely than males to have methylation positive colorectal cancer [11]. Though sex effects appear to differ across studies, no single study so far has focused on the sex effect across the genome.

With the microarray genotyping technique, genome wide methylation measurements are available with up to over 27,578 genetic sites covering 23 pairs of chromosomes (Illumina Infinium Methylation Assay). Such technology will surely lead to genome wide association studies (GWAS) of methylation on various phenotypes. To date, more GWAS have been done on single nucleotide polymorphisms [12] than on methylation. Before conducting GWAS on methylation, one of the key questions that needs to be addressed is how sex influences the genomic methylation status and how to cope with it in the context of methylation-disease association study. Methylation can potentially link to sex and any other phenotypes. As reported by Wu et al. in a lung cancer study, O-6-methylguanine-DNA methyltransferase (*MGMT*) hypermethylation is more common in squamous cell carcinomas in males and smokers than in adenocarcinomas in females, and nonsmokers. *MGMT* hypermethylation was pronouncedly influenced by sex in addition to smoking status [13]. Such results emphasize the need for a proper way to correct the sex influence in a methylation-disease study.

In this paper, we use a subsample (n = 197, 54 female and 143 male) of a larger genome-wide study, which is designed to investigate the association between genomic DNA methylation and alcohol dependence. All participants have some level of substance abuse, and their substance use behavior was assessed through questionnaires. Saliva samples were collected from each participant to extract DNA and then to examine the methylation value using the Illumina Infinium Methylation Assay. The samples were processed in a random order, and the subsamples used in this paper are the first 197 processed samples. We use this data to investigate the sex effect systemically and also propose a correction method to eliminate the potentially confounding effect of sex on methylation-disease association studies. A third-party DNA methylation data from peripheral blood cells assessed by Illumina cancer panel array were used to verify the findings.

## Results

Five behavioral variables as well as age and sex were used as phenotypes to investigate the association with methylation status. The behavioral variables were assessed through self-report questionnaires, described as follows: 1) The Alcohol Use Disorders Identification Test (AUDIT) [14]. In this measure, participants were asked to report the quantity and frequency of heavy drinking and other symptoms associated with alcohol abuse. The items are summed to create a total AUDIT score. 2) The Alcohol Dependence Scale (ADS) [15] includes 24 items with four subscales. The total score was used as the phenotype in this study. 3) Participants were asked to report the maximum number of drinks in a single drinking episode (Max_drinks). 4) Self reported number of cigarettes smoked per day on average (cigarettes use). 5) The percentage of days smoked marijuana in the past 90 days (%_MJ_days).

### Direct association test on each methylation site with phenotypes

**Figure 1:**
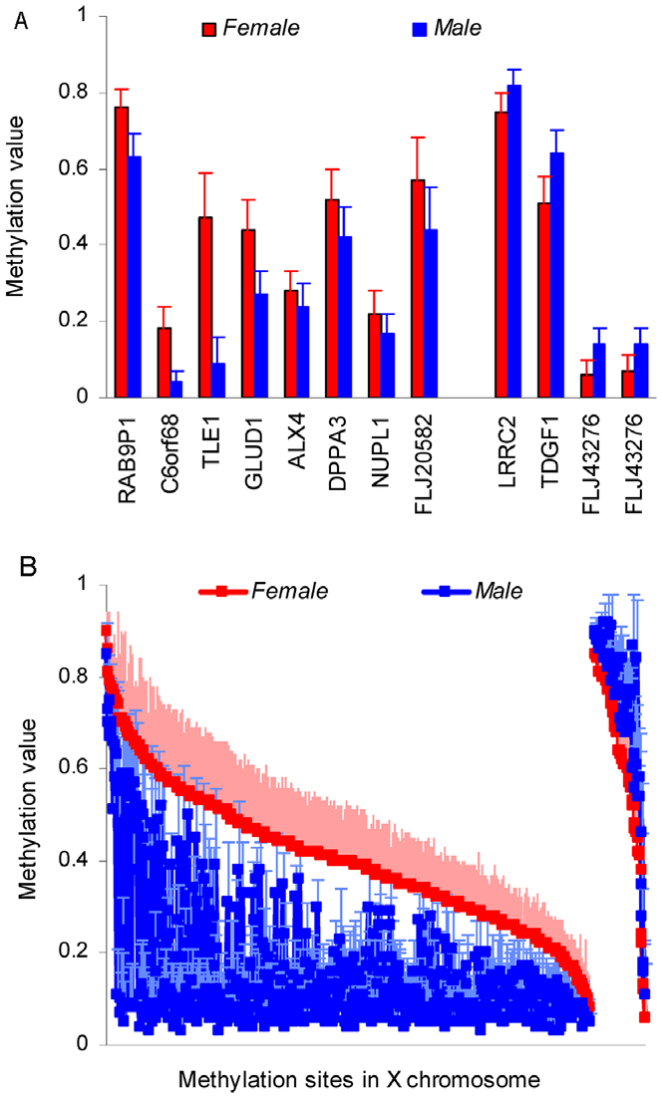
Significant sex effects on 690 methylation sites with mean and standard deviation values. a): mean and standard deviation methylation values from 12 autosomal sites. Red indicates females and blue indicates males. Bars present mean value, while lines show standard deviation. Eight sites are more methylated in females than males and four sites are more methylated in males than females. b): methylation pattern of 678 sites in X chromosome. They are sorted by female methylation level, presenting 614 sites with higher methylation in females and 64 sites with higher methylation in males. Solid squares show mean value while dash lines show standard deviation.

We first examined the sex influence on methylation via a two-sample T-test. The results from genomic 20,493 CpG sites spread out widely, and P-values range from 2.84E-80 to 1. A 5% Bonferroni multiple comparison corrected false positive rate was used to select significant sex effects. Thus, 690 CpG sites in 432 genes were significantly associated with sex (more detailed information see the supporting Table S1). Among the 432 genes, eleven genes including 12 sites are in autosomes: *LRRC2* and *TDGF1* in Chromosome 3, *RAB9P1* in chromosome 5, *C6ORF68* in Chromosome 6; *TLE1* in Chromosome 9, *GLUD1* in Chromosome 10, *ALX4* in Chromosome 11, *DPPA3* in Chromosome 12, *NUPL1* in Chromosome 13, *FLJ20582* and *FLJ43276* in Chromosome 15. The remaining 421 genes including 678 sites are located in the X chromosome. Figure 1A shows methylation values of 12 autosomal sites, while Figure 1B shows the methylation values of sites in the X chromosome with red for females and blue for males.

**Figure 2:**
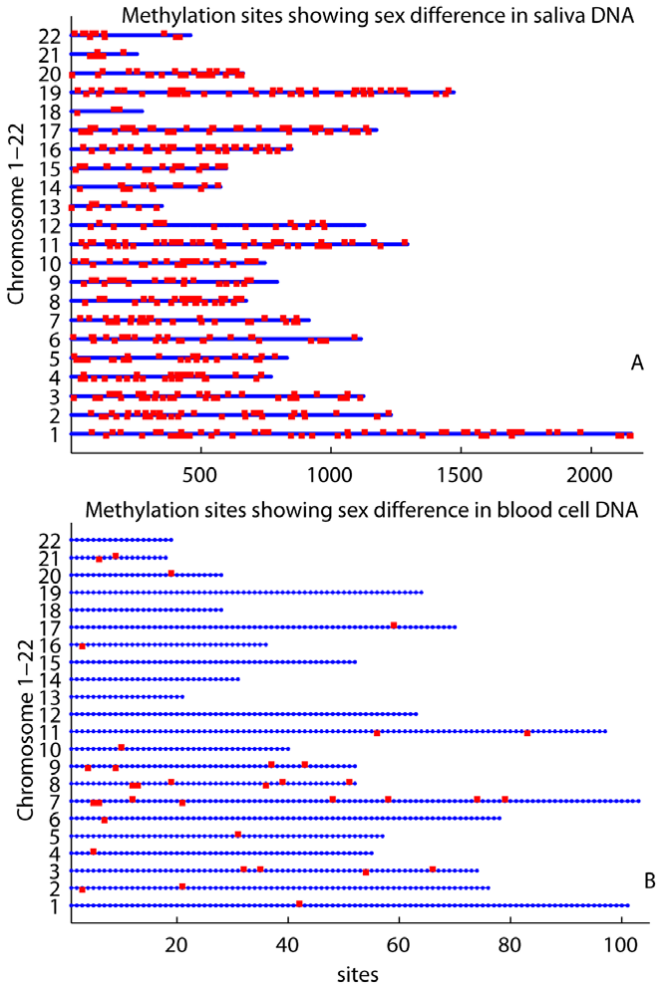
Autosomal sites identified as sex differentially methylated. Human chromosomes 1-22 are arranged vertically. Methylation sites in each chromosome are plotted horizontally in blue line. Red dots present sites showing sex difference based 5% uncorrected false positive rate. The ones above blue lines are sites where females are more methylated than males. The ones below blue lines are sites where males are more methylated than females. a): result from saliva methylation data. 307 sites are methylated more in females than in males, while 273 sites are methylated more in males. b): results from blood cell verification data. 21 sites are methylated more in females than in males, while 15 sites are methylated more in males.

Focusing on the sex influence on autosomes, we found 580 methylation sites (540 genes) in Chromosomes 1 to 22, passing 5% uncorrected false positive rate. Figure 2A shows the locations of such sites on chromosomes (see the supporting Table S2 for the full report), where the top 12 sites passed Bonferroni corrected 5% false positive. Furthermore, we have identified eight functional groups prominently presented in these 540 genes. The eight groups listed in Table 1 were extracted with the highest clustering stringency, using the built-in gene classification function in the **D**atabase for **A**nnotation, **V**isualization and **I**ntegrated **D**iscovery (DAVID, http://david.abcc.ncifcrf.gov) bioinformatics resources [16], [17].

**Table 1:** Functional clusters of the autosomal sex differentially methylated genes.

Gene groups	Enrichment score	Gene List Size	Min P-Value
regulation of transcription, DNA-dependent, DNA-binding, nucleobase, nucleoside, nucleotide and nucleic acid metabolic process	2.23	52	1.8E-38
spliceosome, RNA/mRNA splicing, RNA/mRNA processing, RNA–binding	1.7	5	1.7E-09
signal, trans-membrane, membrane, integral to membrane, intrinsic to membrane	1.27	5	7.6E-04
cadherin, cell-cell adhesion, calcium,	1.27	6	5.5E-13
cation channel activity, metal ion transporter activity, channel activity	1.25	7	1.4E-11
G-protein coupled receptor, transducer, rhodopsin-like G-protein coupled receptor, transmembrane receptor activity	1.03	15	2.5E-19
tyrosine-protein kinase, transmembrane receptor protein tyrosine kinase activity	1.01	5	2.5E-10
protein kinase activity, protein amino acid phosphorylation, protein serine/threonine kinase activity,	0.81	11	1.1E-14

Note: Enrichment score ranks the biological significance of gene groups based on overall modified P-values of all enriched annotation terms.

We also tested associations between methylation and other phenotypes using Pearson correlation. Results were corrected by 5% Bonferroni correction. The correlation coefficient for each phenotype, either significant or not were reported in Table 2 for complete information. Sex, age, max_drinks, and marihuana use show very strong connections with methylation level of specific sites. 85 CpG sites with one in X chromosome were associated with age significantly. Eight sites were significantly related with marijuana use assessment, and none are from Chromosome X. Two sites in gene *PAGE4* and *GRM3* locating in Chromosome X and 5, respectively, were associated with max_drinks level. One site in gene *SEC31L2* in Chromosome 10 was associated with cigarette use. The detailed information about the associated methylation sites were reported in the supporting Table S3.

**Table 2:** Association test results between methylation and phenotypes.

phenotypes	Minimun P-value	Maximum absolute Correlation	Sites passing 5% Borferroni multple comparison corretion
**Before sex effect correction**			
Sex	2.84E-80	32.33 (T value)	690 sites
ADS	2.05E-5	0.30	0
AUDIT	1.10E-5	0.31	0
Max_drinks	1.50E-7	0.36	2 sites
Cigarettes	1.20E-6	0.34	1 site
Age	1.32E-16	0.54	85 sites
%_MJ_days	3.78E-07	0.35	8 sites
**After sex effect correction**			
Sex	1.37E-2	2.48 (T value)	0
ADS	2.07E-5	0.30	0
AUDIT	1.09E-5	0.31	0
Max_drinks	2.67E-7	0.36	2 sites *(2 sites in common* * *)*
Cigarettes	1.41E-6	0.34	1 site *(1 sites in common* * *)*
Age	*1.57E-16*	*0.54*	*85 sites (83 sites in common* * *)*
%_MJ_days	*1.54E-7*	*0.35*	*11 sites (8 sites in common* * *)*

*Common sites before and after sex effect correction.

### PCA methylation factors and their association with phenotypes

Twenty-six factors were extracted from methylation data using principle component analysis (PCA), maintaining 99% of the data variance. A factor here represents a combined effect from multiple methylation sites with various levels of contribution. Two resultant factors show significant sex differences, with P-values of 2.53E-69 and 1.82E-3 passing 5% Bonferroni correction. These two factors are also tested for correlation with other phenotypes and results are listed in Table 3.

**Table 3:** Properties of the sex-related factors extracted by PCA and ICA.

		Sex	ADS	AUDIT	Max_ drinks	Cigarettes	Age	%_ MJ
PCA: Factor 1	Correlation	27.62*	0.11	0.03	0.08	0.01	0.01	0.11
	P-value	2.53E-69	0.12	0.63	0.27	0.93	0.91	0.11
PCA: Factor 2	Correlation	3.16*	0.08	**0.14**	0.08	**0.16**	0.11	0.07
	P-value	1.82E-3	0.26	**0.05**	0.28	**0.02**	0.12	0.34
ICA: Factor 1	Correlation	31.87	0.09	0.00	0.09	0.04	0.03	0.11
	P-value	3.06E-79	0.20	0.95	0.21	0.55	0.64	0.13

Note: bold indicates marginal significant correlations passing 5% uncorrected false positive control. * notes for t values from the two-sample T-test.

### ICA methylation factors and their association with phenotypes

**Figure 3:**
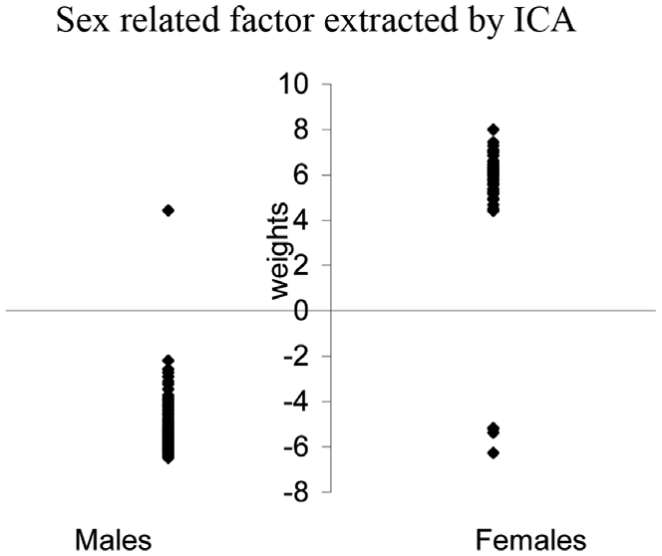
Weights of the sex-related factor expressed in subjects. Weights in 143 males are plotted on the left with two subjects above zeros; weights in 54 females are plotted in the right with four subjects below zero.

Similarly, 26 independent factors were extracted using independent component analysis (ICA), maintaining the same 99% of variance of data. Only one factor is significantly related to sex with a P-value of 3.06E-79. The expression of this factor in subjects is plotted in Figure 3. Females in general have positive expression weights and males have negative weights except for six subjects (two males show positive weight values and four females show negative values). We also tested the correlation of this factor with other phenotypes in Table 3, and no correlation was found significant.

In this sex related factor, each site contributes differently with |Z| score ranging from 83.65 to 0. Z score represents contribution weight of each site to the factor, with positive/negative sign showing the direction. Z score distribution reflects the P-value's distribution derived in the two-sample T-test on sex. Top sites in |Z| scores consist of genes in X chromosome and autosomes, such as *TLE1, GLUD1, C6orf68, RAB9P1, TDGF1, FLJ20582, DPPA3, BXDC1 and LRRC2*, which are consistent with the T-test results. This factor was identified as the sex factor, and we corrected for it using Equation 4 in Materials and Methods section.

### Association of sex-corrected methylation with phenotypes

The association result between methylation sites and phenotypes after the sex effect correction is listed in Table 2. For maximum drink level and cigarette use, the same associations from genes *PAGE4*, *GRM6* and *SEC31L2* were identified. For marijuana use, three additional sites from genes *TCEAL8*, *TIMM8A* and *NOV* demonstrated significant correlations. Two different sites were associated with age after sex effect correction. The details are reported in the supporting Table S3.

### Verification using an independent cancer panel methylation data

The verification data are genomic (1298 loci, 762 genes) methylation values of peripheral blood cell DNA, assessed by Illumina GoldenGate Methylation Cancer Panel I. The two- sample T-test on sex difference identified 47 sites in 26 genes passing 5% Bonferroni correction. Among them, 25 genes are in X chromosome with one site showing higher methylation in males than females and others showing the opposite pattern. The P-value ranges from 1.01E-18 to 2.40E-05. One gene, *CASP6* in Chromosome 4 also presents significant sex difference with female methylation of 0.06±0.02 and male methylation of 0.01±0.01 (P-value: 1.74E-6). Compared with the findings derived from our saliva methylation data, 22 genes demonstrated significant sex difference in both saliva samples and peripheral blood cell samples (see Table S1). Since only 27 sex significantly differentially methylated genes (5% corrected) in saliva (all in X chromosome) are included in the Illumina Cancer Panel I array, the overlapping rate of identified sex affected genes in X chromosome between saliva and blood cell is 81% (22/27).

Focusing on genes in autosomes, we have identified 36 sites (in 34 genes) showing sex differences by the two-sample T-test of 5% uncorrected false positive rate. 21 sites show higher methylation in females than males, and 15 sites show opposite pattern. The distribution of these 36 sites is plotted in Figure 2B in line with the results from saliva samples. Four genes, *MYLK*, *HOXA9*, *PEG10*, and *CDKN2B* present the same strong sex difference in both saliva and blood cell DNA methylation. Since a total of 52 genes in the 540 sex strongly differentially methylated autosomal genes (5% uncorrected) are included in the Illumina Cancer Panel I array, the overlapping rate of autosomal genes showing sex different methylation is 8% (4/52).

ICA was also performed on the verification data using the same parameter setting as on the saliva data. Five independent factors were extracted to keep 99% of the total variance. One factor is significantly related to sex with a P-value of 1.41E-18. All females show positive weights in this factor, averaged at 0.18±0.04. All males show negative weights averaged at −0.13±0.02. After the correction based on the ICA factor, no methylation site shows a sex difference.

## Discussion

Three methods, a direct T-test at each site, PCA and ICA factorization tests all show strong sex influence in the genome wide methylation data from saliva, with the minimum P-values of 2.84E-80, 2.53E-69 and 3.06E-79 respectively. We used a 5% corrected false positive rate to select 690 sites. Their methylation values in Figure 1 show cases in which methylation is higher in females than males or vice verse. It suggests that the influence of sex on methylation is site specific. The presence of more sites with higher methylation in females than males on the X chromosome can be explained by the X-inactivation process, in which one of two copies of genes on the X chromosome in females is silenced. We do not know any mechanism to explain those X chromosome sites with higher methylation in males, but they are very interesting and merit further investigation. In autosomes, our data show a trend of more sites being highly methylated in females versus males; i.e. 8 significant sites verse 3 sites in Figure 1A and 307 sites verse 273 sites in Figure 2A. Fuke et al. discovered subtle but significantly higher 5-methyldeoxycytidine content in males by a high performance liquid chromatography (HPLC) study [18]. The difference, however, might just be caused by different measuring techniques. HPLC is designed to measure the global methylation content without knowing/distinguishing individual sites. Microarray technique using bisulfite measures for specific sites resulting in a methylation level for each site.

Our data, overall, show remarkable sex influences on specific autosomal sites. Genes like *TLE1, C6orf68, GLUD1, RAB9P1, TDGF1, FLG43276, DPPA3, and LRRC2* are the top sex-affected sites that survived stringent Bonferroni correction. For the 580 autosomal sites in Figure 2A, showing strong yet not quite statistically significant sex differences (5% uncorrected), may also reflect true methylation influence from sex. Kaminsky et al. proposed the hypothesis that sex hormone might induce epigenetic change, which predisposes female and male differently to un-Mendelian complex diseases [19]. For example, *TLE1*, a transcriptional repressor essential in hematopoesis, neuronal differentiation and terminal epithelial differentiation, has been shown to contribute to acute myeloid leukemia (AML), synovial sarcoma, and other cancers [20], [21], [22]. Even though the pathways of *TLE1* affecting cancer growth are not clear, our data reflect that the strong sex difference of *TLE1* methylation might explain partially that AML and synovial sarcoma are a little more common in males than females. In the total of 540 autosomal sex differentially affected genes, eight functional groups including many pathways are involved, from DNA transcription, RNA splicing, membrane, trans-membrane, cell to cell adhesion, to ion transporter, etc. It suggests that the influence of sex on methylation is broad and not limited only to sex specific factors.

In our verification analysis, blood cell DNA showed a pattern of methylation which was consistent with what we observed for saliva DNA. Twenty-five genes on the X chromosome and one gene, *CASP6*, on Chromosome 4 were identified a significantly sex difference and the difference occurs in both directions. The overlapping rate of identified genes with saliva data is 81%. Since all overlapped genes are from X chromosome, the ratio presents an attribute of X chromosome between different tissues. When focusing on autosomes, Figure 2 shows that sex influences methylation in a very similar way in both saliva and blood cell DNA (Figure 2A and 2B). Among the 36 autosomal sites showing clear sex difference (5% uncorrected) in blood cells, 21 are highly methylated in females and 15 in males. Four genes show the same sex difference pattern in both saliva and blood cells. The overlapping rate of 8% might be due to the small number of samples and tissue differences. Overall, we are more convinced that sex differences in autosomes may not be statistically significant, but likely present a true effect of sex.

Both datasets from saliva DNA and blood cell DNA present genomic sex effects on methylation; a large amount of loci from both autosomes and X chromosome are affected by sex, and the influence is in both directions (females highly methylated than males or vice verse). We notice that between different tissues (or different populations) the same or different loci can be affected, but the genomic sex effect pattern holds the same.

Methylation of some genes is possibly modulated by both sex and other factors, such as age, substance use, and diseases. For example, in saliva data, methylation status of genes *XPNPEP2*, *PAGE4, TIMM8A* and *TCEAL8* is associated with age, cigarette use and max_drinks, respectively, in addition to sex. Most of the genes in the verification data are functionally cancer related including the genes showing sex difference [23], [24], [25], [26] Both genes *CASP6* and *HOXA9* are hypermethylated, leading to decreased gene expression in pancreas cancer tissue [24]. Gene *PEG10* is involved in human hepatocellular carcinogenesis [26]. Gene *CDKN2B* functionally influences tumor suppression and its methylation has been studied for its power in leukemia treatment [25], [27], [28]. For studies of genetic function or formation of diseases, the sex influence can potentially obscure the methylation–disease association results. It is thus important to take into account the sex confounding effect. There are at least three different ways to address the sex effect. First, we can use sites only from autosomes. Such an approach is simple and most likely effective, but some important information might be lost. As shown in this study, *PAGE4*, *XPNPEP2, TCEAL8*, and *TIMM8A* from Chromosome X contribute greatly to non-sex phenotypes. Second, we can try to remove sites associated with sex only, but that requires a threshold setting to select sex-related sites. This approach will be subjective and likely suboptimal. Finally, we can try to correct the sex effect using factorization methods. Sex factor(s) can encompass both strong and weak influences on multiple sites, without selecting an arbitrary cut-off point. Then we can correct the sex effect by removing the factor(s). PCA and ICA are two such factorization methods which were compared in this study.

Two factors extracted from the PCA show a significant association with sex, while ICA extracted only one strong sex related factor. Since both PCA and ICA maintain the same amount of variance in the original data, the extracted factors are representing the same information in a slightly different format. ICA emphasizes the independence of factors, while PCA only considers the orthogonality of factors. It is likely that ICA is able to pull the information of the two PCA sex related factors into one factor based on the independence criterion. Table 3 also shows that the sex related factor by ICA is not associated with any other phenotypes of interest, while the 2^nd^ factor by PCA shows marginal connection with AUDIT and cigarette use. This is strong evidence that suggests we should utilize the sex factor from ICA not from PCA results, since the correction will not jeopardize the associations of other known phenotypes. The Z scores of the sex related factor by ICA displays a similar pattern to P-values of the T-test on each site. This result provides additional evidence that the ICA sex factor represents the true sex influence accommodating both strong to weak effects. Figure 3 displays how the ICA sex factor is revealed in subjects, with each subject manifesting the sex factor with an individual expression weight. The sex factor represents the common sex influence trend extracted from all subjects, but its expression level allows individual difference. The weights are clearly clustered into two groups: positive and negative. Most of the females show positive weights and most of the males show negative weights. We do not know exactly why six subjects are showing the opposite pattern, but we hypothesize that individual endocrinological differences might be involved [19]. ICA is also able to extract one sex related factor from the verification data, where all females have positive expression weights and males have negative expression weights.

Sex correction was performed based on the ICA factor, and Table 2 compares the results of the direct correlation test before and after the correction. All phenotypes, except sex, show very similar results. After the correction two different sites out of 85 are linked to age, and three additional sites are added to be associated to %_MJ_days. The similarity tells us that sex correction, as expected, removes effects only from sex and no harm is done for sex irrelevant sites. More importantly, the fact that more sites show significant relationships, indicates that sex can be a confounding factor, influencing association analysis of some methylation sites, and more accurate associations can be determined after the correction.

This study focuses on the sex influence on genomic methylation and correction. Strong evidences from both saliva data and blood cell verification data show that sex affects methylation both genome wide and site-specific. A higher methylation in females or in males can occur, and can occur in autosomes and the X chromosome. The strong, yet not statistically significant sex differences in autosomes may truly represent the influence of sex. Moreover, sex effects can be entangled with other environmental factors. We proposed a simple method to disentangle and correct the sex effect. ICA is a data driven method to extract factors based on high order statistics of data. Given different datasets, e.g. methylation from different tissues, the sex factor extracted by ICA can be different in terms of loci and influence strength, but it should be significantly correlated with sex. Therefore, sex effect correction is not a fix value correction, instead, adapted to the data. Confirmation of the significant association with sex is necessary to identify the sex factor. An alternative approach would be to simply analyze female subjects and male subjects separately. This may be a good approach, when the sample size is big enough to divide into two groups. However, in genome wide studies, we usually face a situation of large dimensionality and small sample size, and maximizing sample size in the analysis is always desirable. Besides, some phenotypes like cancer have different incidence rates in females and males, and it is important to identify the underlying mechanisms. The sex factor extracted by ICA and its expression on each individual provide a direct metric to explain the sex influence and quantify the influence on each individual. A sex correction method based on factorization is able to account for both strong and weak sex effects without drawing a cutoff line, which is most likely reflecting how sex influences methylation status on different sites. We have demonstrated that in this study the sex factor is not related to any other known phenotypes, but there is the possibility that some unknown trait might contribute to the observed sex difference, and the correction would negatively impact the analysis. For such a case, the researcher should measure the trait first, and test whether it is related to the sex factor. If not, the correction can proceed. Otherwise, a different approach should be used. Every methylation site showing a significant association with any phenotype is of great interest, and in this study we are just providing a reference point for further investigation. When we have a relatively small set of interesting loci, verification by pyrosequencing or a single nucleotide primer extension based assay will be very helpful.

## Materials and Methods

### Subjects

One hundred ninety-seven participants including 54 females with age 32.02±10.73 and 143 males with age 32.22±9.70 were investigated in this study, which is a subsample of an on-going study designed to investigate genetic prediction for substance dependence. Subjects between age 21 and 55 with a minimum alcohol consumption of a regular pattern of two binge drinking episodes per week (allowing comorbidity of tobacco and marijuana use), otherwise healthy (no history of severe brain injure or brain related medical problems, no symptoms of psychosis during a diagnostic interview), were included. The behavioral variables were assessed through self-report questionnaires during the interview. The samples were processed in a random order, and the subsamples used in this paper are the first 197 processed samples, including treatment-seeking and non-treatment-seeking participants. Participants obtain a wide range of alcohol use severity (AUDIT range  = 6–38) in order to better assess the relationship between genetics and drinking problems. The study was conducted according to the principles expressed in the Declaration of Helsinki, approved by the Institutional Review Board of University of New Mexico. All patients provided written informed consent for the collection of samples and subsequent analysis.

### Methylation measurement

Participants were instructed to deliver 5 ml of saliva into a sterile 50 ml conical centrifuge tube. DNA was then extracted from saliva, purified, bisulfite converted and hybridized. The Illumina Infinium Methylation Assay was used to detect genome wide 27,578 CpG sites, spanning 14,495 genes. The CpG sites locate within the proximal promoter regions of genes, with distance to transcription start site ranging from 0 to 1499 bp averaged at 389±341 bp. A methylation value was outputted for each site, which is a continuous variable between 0 and 1, representing the ratio of the intensity of the methy lated type to the total intensity. Zero means no methylation, and one means 100% methylation. The reproducibility of Illumina assay methylation is reported as R^2^ of 0.98, and the standard deviation of methylation values from replicates is less than 0.06 [29]. We observed a very similar property in our data using two replicates test (R^2^ = 0.97, SD = 0.064). Among the 27,578 CpG sites, some sites have shown either low level averaged methylation or low level variation among all 197 subjects. They thus convey very limited information for further study and great influence of measurement errors. We eliminated these sites using an empirical threshold setting of averaged methylation being 5% of maximum value, and/or variance being 1% of maximum variation. This results in 20,493 CpG sites for further study.

### Analyses methods

We use three different ways to test the connection between sex and genome-wide methylation status, as well as its potential influence or confounding effects on associations of methylation with other phenotypes. Furthermore, a sex effect correction method was introduced based on factorization, and comparisons before and after the sex effect correction were conducted.

Firstly, a two-sample T-test on sex was performed on each methylation site, and Pearson correlation was performed to test association of methylation with other phenotypes. All results are corrected using 5% Bonferroni multiple comparison correction. The significant associations and/or the associations with minimum P-values were listed for each phenotype. When focusing autosome methylation, we chose 5% uncorrected P-value to identify all possible sites affected by sex. Then, we grouped these autosomal sex affected genes based on their functional similarity. DAVID gene functional classification tool (http://david.abcc.ncifcrf.gov/) was used due to its ability to provide a rapid means to organize large lists of genes into functionally related groups to help unravel the biological content captured by high throughput technologies [16], [17]. Being conservative, we used the highest classification stringency to extract the most reliable gene groups.

Secondly, PCA, a factorization method, was utilized to extract factors explaining maximum variance of data. The generic formula of PCA is presented as [30]:



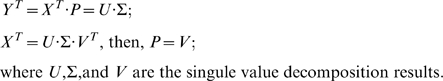
(1)



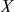
 is the observation data with each row representing a repetition of the experiment and each column representing a variable or a dimension. In this study 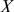 is the methylation measurement from each subject. 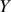
is the new representation of data–the latent principle components/factors. 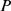 is the principle component coefficient, carrying each factor's expression pattern in subjects. PCA projects data into new directions so that each direction is orthogonal to each other. We then test the connection of 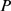 with sex and other phenotypes, aiming at identifying the sex factor.

Similar to PCA, ICA is also used to extract hidden factors in the data, but through higher order statistics enabling maximization of the independence of each factor. ICA is generally formed as:
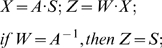
(2)

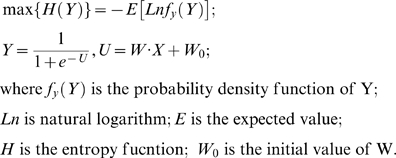
(3)


*X* is an observation data matrix that can be composed of measurements such as speech signals from multiple microphones, or subject's methylation values. *S* contains the independent components, which consists of unknown sources such as multiple speakers' voices, or methylation factors accounting for various phenotypes. *A* is a linear mixing matrix, relating the sources to the mixed measurements. *W* is an unmixing matrix. If *W* equals the inverse of *A*, then the *Z*, the estimated component matrix, is equivalent to *S*, the source matrix. Therefore, the essence of ICA is to find *W* so that *Z* is as close as possible to the true independent components contained in *S*. There are many ICA algorithms based on different independence criteria. Among them, the infomax algorithm attempts to find the *W* matrix through maximizing an entropy function as defined in Equation 3 [31], [32]. We use infomax to extract factors/components, and then tested their associations with sex and other phenotypes.

Thirdly, a factor significantly associated with only sex and not other phenotypes is defined as a sex factor. The effect of this factor is removed from the observation data using the Equation 4, where 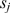 is the identified sex factor. The corrected data 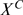 has the same dimension as 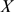, containing all the methylation sites corrected for sex.
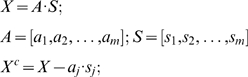
(4)


Finally, the two-sample T-test on sex and Pearson correlation between methylation and other phenotypes was tested again after the sex effect correction.

### Verification data and methods

To verify the findings of this study, we chose a third-party independent methylation data (GEO accession: GSE19515), and conducted the same analysis. The GSE19515 dataset was uploaded by the German cancer research center, including genomic methylation data of 27 samples, as well as their sex information. In this dataset, DNA was prepared from peripheral blood cells of 1 patient (at time of diagnosis and month 27) and 25 healthy controls (12 males and 13 females). The Ilumina GoldenGate Methylation Cancer Panel I array was used, spanning 1,505 CpG sites from promoter regions or the first exon of 808 mostly cancer related genes (based on gene annotation). To be consistent, we only use 25 healthy controls data in the verification process. Data quality control was first applied. Two loci (DAB2IP_P671_F and SCGB3A1_P103_R) from genes DAB2IP and SCGB3A1 were removed due to their high averaged detection P-values (>0.5, i.e. targeted signal is lower than 50% of negative control signals). Two hundred and five loci were removed from analysis due to low level methylation or low variance (averaged methylation value <0.01, or averaged variance <0.0001). Thus, the verification data include 25 healthy subjects and 1298 loci (762 genes, 39 of them from X chromosome). Among these 762 genes, 672 genes are included in our saliva DNA methylation data. We first conducted a two-sample T-test on methylation of each locus regarding sex difference, and corrected the results with 5% Bonferroni correction. Then, we used an uncorrected 5% P-value to select all autosomal loci showing sex differences. Finally, we performed the ICA analysis to identify the sex related factor. All results were compared with the corresponding findings from our saliva data.

## Supporting Information

Table S1 960 sites showing significant sex difference on saliva DNA methylation. 960 sites from autosomes and X chromosome showing significant sex difference on saliva DNA methylation. Some also show significant sex difference on peripheral blood cell DNA methylation.
(0.45 MB DOC)

Table S2 580 autosomal sites showing sex difference on saliva DNA methylation. Some also show sex difference on peripheral blood cell DNA methylation.
(0.66 MB DOC)

Table S3 Correlation results before and after sex effect correction. Significant correlation of methylation with age, marijuana use, maximum drink, and cigarette use before and after sex effect correction.
(0.14 MB DOC)

## References

[1] Tost J, Tost J (2008). DNA Methylation: An Introduction to the Biology and the Disease-Associated Changes of a Promising Biomarker.. DNA Methylation: Methods and Protocols. 2 ed.

[2] Philibert RA, Gunter TD, Beach SR, Brody GH, Madan A (2008). MAOA methylation is associated with nicotine and alcohol dependence in women.. Am J Med Genet B Neuropsychiatr Genet.

[3] Nagarajan RP, Hogart AR, Gwye Y, Martin MR, LaSalle JM (2006). Reduced MeCP2 expression is frequent in autism frontal cortex and correlates with aberrant MECP2 promoter methylation.. Epigenetics.

[4] Abdolmaleky HM, Cheng KH, Faraone SV, Wilcox M, Glatt SJ (2006). Hypomethylation of MB-COMT promoter is a major risk factor for schizophrenia and bipolar disorder.. Hum Mol Genet.

[5] McGowan PO, Sasaki A, D'Alessio AC, Dymov S, Labonte B (2009). Epigenetic regulation of the glucocorticoid receptor in human brain associates with childhood abuse.. Nat Neurosci.

[6] Rakyan VK, Hildmann T, Novik KL, Lewin J, Tost J (2004). DNA methylation profiling of the human major histocompatibility complex: a pilot study for the human epigenome project.. PLoS Biol.

[7] Eckhardt F, Lewin J, Cortese R, Rakyan VK, Attwood J (2006). DNA methylation profiling of human chromosomes 6, 20 and 22.. Nat Genet.

[8] Sarter B, Long TI, Tsong WH, Koh WP, Yu MC (2005). Sex differential in methylation patterns of selected genes in Singapore Chinese.. Hum Genet.

[9] El-Maarri O, Becker T, Junen J, Manzoor SS, Diaz-Lacava A (2007). Gender specific differences in levels of DNA methylation at selected loci from human total blood: a tendency toward higher methylation levels in males.. Hum Genet.

[10] Xiao Y, Word B, Starlard-Davenport A, Haefele A, Lyn-Cook BD (2008). Age and gender affect DNMT3a and DNMT3b expression in human liver.. Cell Biol Toxicol.

[11] Wiencke JK, Zheng S, Lafuente A, Lafuente MJ, Grudzen C (1999). Aberrant methylation of p16INK4a in anatomic and gender-specific subtypes of sporadic colorectal cancer.. Cancer Epidemiol Biomarkers Prev.

[12] Liu J, Pearlson G, Windemuth A, Ruano G, Perrone-Bizzozero NI (2009). Combining fMRI and SNP data to investigate connections between brain function and genetics using parallel ICA.. Hum Brain Mapp.

[13] Wu J-Y, Wang J, Lai J-C, Cheng Y-W, Yeh K-T (2008). Association of O6-Methylguanine-DNA Methyltransferase (MGMT) Promoter Methylation with p53 Mutation Occurrence in Non-Small Cell Lung Cancer with Different Histology, Gender, and Smoking Status.. Ann Surg Oncol.

[14] Babor T, Higgins-Biddle JC, Saunders JB, Monteiro MG (2006). AUDIT: Alcohol Use Disorders Identification Test: guidelines for use in primary care..

[15] Skinner HA, Allen BA (1982). Alcohol dependence syndrome: measurement and validation.. J Abnorm Psychol.

[16] Dennis G, Sherman BT, Hosack DA, Yang J, Gao W (2003). DAVID: Database for Annotation, Visualization, and Integrated Discovery.. Genome Biol.

[17] Huang da W, Sherman BT, Lempicki RA (2009). Systematic and integrative analysis of large gene lists using DAVID bioinformatics resources.. Nat Protoc.

[18] Fuke C, Shimabukuro M, Petronis A, Sugimoto J, Oda T (2004). Age related changes in 5-methylcytosine content in human peripheral leukocytes and placentas: an HPLC-based study.. Ann Hum Genet.

[19] Kaminsky Z, Wang SC, Petronis A (2006). Complex disease, gender and epigenetics.. Ann Med.

[20] Fraga MF, Berdasco M, Ballestar E, Ropero S, Lopez-Nieva P (2008). Epigenetic inactivation of the Groucho homologue gene TLE1 in hematologic malignancies.. Cancer Res.

[21] Kosemehmetoglu K, Vrana JA, Folpe AL (2009). TLE1 expression is not specific for synovial sarcoma: a whole section study of 163 soft tissue and bone neoplasms.. Mod Pathol.

[22] Terry J, Saito T, Subramanian S, Ruttan C, Antonescu CR (2007). TLE1 as a diagnostic immunohistochemical marker for synovial sarcoma emerging from gene expression profiling studies.. Am J Surg Pathol.

[23] Das PM, Ramachandran K, Vanwert J, Ferdinand L, Gopisetty G (2006). Methylation mediated silencing of TMS1/ASC gene in prostate cancer.. Mol Cancer.

[24] Tan AC, Jimeno A, Lin SH, Wheelhouse J, Chan F (2009). Characterizing DNA methylation patterns in pancreatic cancer genome.. Mol Oncol.

[25] Raj K, John A, Ho A, Chronis C, Khan S (2007). CDKN2B methylation status and isolated chromosome 7 abnormalities predict responses to treatment with 5-azacytidine.. Leukemia.

[26] Okabe H, Satoh S, Furukawa Y, Kato T, Hasegawa S (2003). Involvement of PEG10 in Human Hepatocellular Carcinogenesis through Interaction with SIAH1.. Cancer Res.

[27] Au W-Y, Fung AT, Ma ES, Chan C-H, Wong K-F (2005). Serial studies of methylation of CDKN2B and CDKN2A in relapsed acute promyelocytic leukaemia treated with arsenic trioxide.. British Journal of Haematology.

[28] Chim CS, Lau JS, Wong KF, Kwong YL (2005). CDKN2B methylation is an independent poor prognostic factor in newly diagnosed acute promyelocytic leukemia.. Leukemia.

[29] Bibikova M, Lin Z, Zhou L, Chudin E, Garcia EW (2006). High-throughput DNA methylation profiling using universal bead arrays.. Genome Res.

[30] Jackson JE (1991). A User's Guide to Principal Components..

[31] Cardoso JF (1997). Infomax and maximum likelihood for blind source separation.. IEEE Signal Processing Letters.

[32] Bell AJ, Sejnowski TJ (1995). An information-maximization approach to blind separation and blind deconvolution.. Neural Comput.

