# Supplementary material for: A Study of the Influence of Sex on Genome Wide Methylation
Source: PLoS One. 2010 Apr 6;5(4):e10028. doi: 10.1371/journal.pone.0010028 (PMC2850313; doi:10.1371/journal.pone.0010028)
Supplement: Table S1 — 960 sites showing significant sex difference on saliva DNA methylation. 960 sites from autosomes and X chromosome showing significant sex difference on saliva DNA methylation. Some also show significant sex difference on peripheral blood cell DNA methylation. (0.45 MB DOC) [file pone.0010028.s001.doc]

Supporting Table S1. 960 sites showing significant sex difference on saliva DNA methyaltion

| Gene | Target ID | Female  β-value | Male  β-value | P value | Gene | Target ID | Female  β-value | Male  β-value | P value |
| --- | --- | --- | --- | --- | --- | --- | --- | --- | --- |
| BEX1 | cg23106779 | 0.62±0.18 | 0.03±0.08 | 2.84E-80 | FAM58A | cg14346048 | 0.36±0.08 | 0.14±0.07 | 1.75E-47 |
| PORCN | cg08076508 | 0.43±0.12 | 0.05±0.05 | 3.20E-79 | ESX1 | cg04297329 | 0.33±0.12 | 0.08±0.07 | 1.86E-47 |
| ELK1 | cg21195120 | 0.65±0.19 | 0.04±0.08 | 3.54E-79 | RAB39B | cg00078867 | 0.25±0.07 | 0.08±0.05 | 2.14E-47 |
| DXS9879E | cg19928247 | 0.62±0.16 | 0.07±0.08 | 1.11E-78 | SYP | cg15536552 | 0.4±0.09 | 0.16±0.07 | 3.09E-47 |
| SLC9A6 | cg07980988 | 0.71±0.19 | 0.07±0.09 | 1.60E-78 | RAP2C | cg08262933 | 0.41±0.11 | 0.13±0.08 | 4.31E-47 |
| IDS | cg08420900 | 0.52±0.14 | 0.06±0.06 | 2.15E-78 | PNCK | cg12449183 | 0.4±0.11 | 0.13±0.08 | 5.57E-47 |
| OCRL | cg20849987 | 0.52±0.15 | 0.06±0.06 | 2.38E-78 | SLITRK4 | cg05141400 | 0.27±0.07 | 0.08±0.06 | 5.60E-47 |
| PHF6 | cg15677355 | 0.44±0.13 | 0.05±0.05 | 2.46E-78 | WWC3 | cg24496423 | 0.31±0.1 | 0.09±0.06 | 5.74E-47 |
| TMSL8 | cg18473117 | 0.61±0.17 | 0.05±0.08 | 2.63E-78 | STAG2 | cg25410279 | 0.42±0.1 | 0.18±0.07 | 6.07E-47 |
| LANCL3 | cg01215061 | 0.41±0.11 | 0.05±0.05 | 3.98E-78 | LONRF3 | cg20641280 | 0.35±0.09 | 0.12±0.07 | 7.21E-47 |
| BCORL1 | cg17889912 | 0.4±0.12 | 0.04±0.05 | 4.19E-78 | TAB3 | cg03122511 | 0.56±0.08 | 0.76±0.06 | 1.12E-46 |
| PHF8 | cg09018040 | 0.53±0.14 | 0.08±0.06 | 4.34E-78 | TMEM32 | cg21229055 | 0.26±0.09 | 0.06±0.05 | 1.50E-46 |
| NDUFA1 | cg13928866 | 0.46±0.13 | 0.06±0.05 | 4.47E-78 | PRAF2 | cg19523692 | 0.42±0.1 | 0.17±0.07 | 2.63E-46 |
| CXorf40A | cg03161453 | 0.57±0.16 | 0.05±0.07 | 4.50E-78 | BCOR | cg02549418 | 0.52±0.13 | 0.87±0.11 | 6.28E-46 |
| PRDX4 | cg06927864 | 0.62±0.17 | 0.06±0.08 | 5.98E-78 | RBMX2 | cg13601079 | 0.4±0.11 | 0.14±0.07 | 1.06E-45 |
| ARMCX4 | cg19392138 | 0.51±0.15 | 0.03±0.07 | 6.17E-78 | ZNF6 | cg23698956 | 0.35±0.09 | 0.12±0.07 | 1.08E-45 |
| MPP1 | cg25248094 | 0.57±0.16 | 0.06±0.07 | 8.65E-78 | ZXDA | cg02020018 | 0.31±0.08 | 0.09±0.07 | 1.10E-45 |
| EBP | cg07874284 | 0.54±0.15 | 0.06±0.07 | 9.08E-78 | AMOT | cg26644504 | 0.3±0.09 | 0.09±0.06 | 1.29E-45 |
| FLJ20298 | cg03164831 | 0.5±0.14 | 0.06±0.06 | 1.18E-77 | DKFZp564K142 | cg15657668 | 0.3±0.1 | 0.07±0.07 | 1.55E-45 |
| EDA | cg19771541 | 0.57±0.16 | 0.04±0.07 | 1.82E-77 | GNL3L | cg26053787 | 0.36±0.11 | 0.12±0.07 | 2.02E-45 |
| MGC39606 | cg16343842 | 0.54±0.15 | 0.06±0.07 | 2.54E-77 | PFKFB1 | cg10634358 | 0.68±0.08 | 0.84±0.04 | 2.88E-45 |
| RBMX | cg11714014 | 0.46±0.13 | 0.07±0.05 | 4.05E-77 | COX7B | cg17694877 | 0.39±0.1 | 0.16±0.07 | 3.49E-45 |
| GPC3 | cg06659128 | 0.56±0.17 | 0.03±0.07 | 4.06E-77 | STARD8 | cg12691219 | 0.57±0.13 | 0.24±0.1 | 5.50E-45 |
| PLP2 | cg11871549 | 0.7±0.2 | 0.05±0.09 | 5.05E-77 | GLA | cg05573563 | 0.39±0.13 | 0.11±0.08 | 9.67E-45 |
| PHKA2 | cg06277838 | 0.54±0.15 | 0.05±0.06 | 5.42E-77 | GLUD2 | cg11472424 | 0.49±0.08 | 0.3±0.06 | 1.01E-44 |
| OPHN1 | cg04287191 | 0.42±0.12 | 0.06±0.05 | 6.92E-77 | ARMCX3 | cg06384491 | 0.2±0.07 | 0.03±0.05 | 1.80E-44 |
| TFE3 | cg00800095 | 0.48±0.14 | 0.05±0.06 | 7.98E-77 | MID1IP1 | cg21224759 | 0.36±0.09 | 0.12±0.08 | 2.27E-44 |
| SCML2 | cg25424556 | 0.47±0.14 | 0.04±0.06 | 8.10E-77 | SOX3 | cg23717696 | 0.26±0.08 | 0.07±0.05 | 3.34E-44 |
| DNASE1L1 | cg08575950 | 0.52±0.14 | 0.08±0.06 | 1.09E-76 | PAK3 | cg14132995 | 0.24±0.07 | 0.06±0.06 | 7.20E-44 |
| EFNB1 | cg15016628 | 0.67±0.18 | 0.09±0.09 | 2.19E-76 | GPR101 | cg00799727 | 0.33±0.1 | 0.07±0.08 | 7.37E-44 |
| PIM2 | cg14774175 | 0.51±0.15 | 0.05±0.06 | 2.42E-76 | MAGEE2 | cg04659622 | 0.57±0.09 | 0.29±0.1 | 9.62E-44 |
| CXorf40A | cg16510010 | 0.58±0.16 | 0.07±0.07 | 3.06E-76 | REPS2 | cg20104776 | 0.35±0.09 | 0.13±0.07 | 1.22E-43 |
| CXorf34 | cg12654845 | 0.61±0.16 | 0.09±0.08 | 5.74E-76 | CXorf41 | cg01961936 | 0.35±0.07 | 0.17±0.06 | 1.44E-43 |
| REPS2 | cg25933726 | 0.5±0.15 | 0.05±0.06 | 8.00E-76 | PGK1 | cg09840989 | 0.5±0.12 | 0.15±0.12 | 2.00E-43 |
| ZIC3 | cg12731488 | 0.51±0.13 | 0.09±0.06 | 1.01E-75 | KIF4A | cg16342780 | 0.35±0.11 | 0.1±0.08 | 2.34E-43 |
| MAGED4 | cg25932752 | 0.45±0.13 | 0.05±0.06 | 1.08E-75 | AMMECR1 | cg27351998 | 0.25±0.06 | 0.11±0.04 | 2.77E-43 |
| SLC10A3 | cg10526888 | 0.6±0.18 | 0.06±0.08 | 1.12E-75 | ARX | cg24340926 | 0.35±0.1 | 0.11±0.07 | 2.95E-43 |
| UBE2A | cg14105781 | 0.54±0.15 | 0.07±0.07 | 1.57E-75 | SRPX | cg22998840 | 0.44±0.1 | 0.17±0.09 | 3.81E-43 |
| MTCP1 | cg23493704 | 0.44±0.14 | 0.05±0.05 | 1.85E-75 | PDHA1 | cg12753358 | 0.4±0.1 | 0.17±0.07 | 5.66E-43 |
| LDOC1 | cg19407886 | 0.59±0.16 | 0.07±0.08 | 1.94E-75 | USP11 | cg15211677 | 0.27±0.09 | 0.1±0.05 | 5.76E-43 |
| FUNDC2 | cg21137707 | 0.48±0.13 | 0.08±0.05 | 1.96E-75 | RNF12 | cg25941716 | 0.53±0.1 | 0.28±0.08 | 6.53E-43 |
| TSPYL2 | cg24054653 | 0.68±0.19 | 0.08±0.09 | 2.03E-75 | CDX4 | cg00690049 | 0.62±0.1 | 0.35±0.09 | 1.74E-42 |
| TAF1 | cg09555679 | 0.51±0.14 | 0.07±0.06 | 2.65E-75 | PDCD8 | cg24522076 | 0.34±0.11 | 0.12±0.07 | 2.24E-42 |
| MASK | cg07917796 | 0.47±0.13 | 0.06±0.06 | 2.87E-75 | BCOR | cg23384620 | 0.46±0.14 | 0.84±0.13 | 2.51E-42 |
| OTUD5 | cg09086179 | 0.57±0.15 | 0.11±0.07 | 2.95E-75 | MAGEA8 | cg04553232 | 0.59±0.09 | 0.77±0.05 | 2.76E-42 |
| CXorf26 | cg24964364 | 0.44±0.11 | 0.07±0.06 | 2.98E-75 | GLUD1 | cg14271499 | 0.44±0.08 | 0.27±0.06 | 4.61E-42 |
| FAM70A | cg03712237 | 0.53±0.14 | 0.08±0.07 | 3.34E-75 | SLC16A2 | cg05673087 | 0.46±0.14 | 0.08±0.14 | 5.94E-42 |
| ZBTB33 | cg02004156 | 0.51±0.14 | 0.08±0.07 | 4.56E-75 | DYNLT3 | cg19410841 | 0.4±0.09 | 0.2±0.07 | 9.12E-42 |
| PDK3 | cg17659886 | 0.55±0.15 | 0.07±0.07 | 4.74E-75 | ZIC3 | cg24648715 | 0.6±0.07 | 0.41±0.07 | 1.19E-41 |
| APEX2 | cg05373692 | 0.39±0.12 | 0.04±0.05 | 5.16E-75 | LANCL3 | cg25100404 | 0.21±0.05 | 0.11±0.03 | 1.29E-41 |
| PGRMC1 | cg07146718 | 0.51±0.15 | 0.06±0.06 | 5.93E-75 | TMEM47 | cg13794642 | 0.36±0.09 | 0.16±0.06 | 1.76E-41 |
| GABRE | cg11552293 | 0.42±0.12 | 0.06±0.05 | 6.60E-75 | TCEAL2 | cg27305895 | 0.3±0.07 | 0.15±0.05 | 3.98E-41 |
| RPGR | cg25316166 | 0.57±0.16 | 0.08±0.07 | 6.73E-75 | BCAP31 | cg01172484 | 0.3±0.1 | 0.08±0.07 | 4.21E-41 |
| MTMR1 | cg23137494 | 0.56±0.14 | 0.1±0.07 | 8.16E-75 | CYSLTR1 | cg26150490 | 0.36±0.09 | 0.15±0.07 | 1.85E-40 |
| POLA | cg13928116 | 0.51±0.13 | 0.09±0.07 | 8.32E-75 | MOSPD1 | cg00145348 | 0.62±0.06 | 0.41±0.08 | 2.61E-40 |
| LAS1L | cg16122592 | 0.44±0.12 | 0.06±0.06 | 1.02E-74 | G6PD | cg18289259 | 0.29±0.1 | 0.08±0.07 | 2.63E-40 |
| AR | cg05113908 | 0.6±0.14 | 0.16±0.07 | 1.17E-74 | MAGED1 | cg00725777 | 0.33±0.07 | 0.13±0.07 | 6.64E-40 |
| IL13RA1 | cg00646140 | 0.61±0.17 | 0.09±0.08 | 1.63E-74 | ZNF673 | cg14520448 | 0.21±0.06 | 0.07±0.05 | 7.29E-40 |
| EMD | cg08591489 | 0.75±0.19 | 0.11±0.1 | 1.63E-74 | ERAS | cg12576145 | 0.54±0.09 | 0.31±0.08 | 1.19E-39 |
| SYP | cg10009830 | 0.62±0.17 | 0.08±0.08 | 1.85E-74 | P2RY4 | cg04222374 | 0.77±0.07 | 0.89±0.03 | 1.36E-39 |
| ZNF41 | cg01135626 | 0.4±0.11 | 0.05±0.05 | 1.94E-74 | SLITRK2 | cg26655138 | 0.19±0.08 | 0.06±0.03 | 1.69E-39 |
| NGFRAP1 | cg05105069 | 0.58±0.15 | 0.09±0.07 | 2.07E-74 | RAP2C | cg14044580 | 0.37±0.1 | 0.16±0.07 | 2.62E-39 |
| FAM11A | cg02504280 | 0.47±0.14 | 0.05±0.06 | 2.24E-74 | MCTS1 | cg21258987 | 0.28±0.1 | 0.07±0.07 | 3.87E-39 |
| MAGED2 | cg00151234 | 0.46±0.11 | 0.1±0.06 | 2.34E-74 | COVA1 | cg20308511 | 0.22±0.07 | 0.08±0.05 | 1.36E-38 |
| SLC9A7 | cg14354749 | 0.48±0.13 | 0.07±0.06 | 2.38E-74 | NAP1L2 | cg03455762 | 0.4±0.09 | 0.17±0.09 | 1.71E-38 |
| ZCCHC12 | cg00673020 | 0.47±0.11 | 0.11±0.06 | 2.95E-74 | FGD1 | cg10894453 | 0.28±0.09 | 0.11±0.05 | 2.87E-38 |
| AR | cg21248478 | 0.45±0.13 | 0.05±0.06 | 3.17E-74 | GLT28D1 | cg19140639 | 0.33±0.12 | 0.08±0.09 | 3.39E-38 |
| MOSPD1 | cg05856884 | 0.49±0.13 | 0.06±0.07 | 4.30E-74 | MAGEE1 | cg04037732 | 0.44±0.08 | 0.24±0.07 | 4.70E-38 |
| ZMYM3 | cg24432916 | 0.48±0.15 | 0.04±0.06 | 4.52E-74 | PCSK1N | cg06900776 | 0.54±0.09 | 0.34±0.07 | 4.94E-38 |
| IGBP1 | cg20063650 | 0.3±0.08 | 0.07±0.03 | 5.01E-74 | PDHA1 | cg02725692 | 0.22±0.06 | 0.08±0.05 | 5.14E-38 |
| WNK3 | cg12064213 | 0.46±0.12 | 0.08±0.06 | 5.34E-74 | RP2 | cg23813564 | 0.39±0.12 | 0.14±0.09 | 5.54E-38 |
| RP13-360B22.2 | cg07177300 | 0.52±0.12 | 0.12±0.06 | 1.07E-73 | TSPYL2 | cg17073891 | 0.38±0.09 | 0.17±0.08 | 7.68E-38 |
| HMGB3 | cg05327750 | 0.65±0.18 | 0.08±0.09 | 1.51E-73 | UXT | cg19407266 | 0.28±0.07 | 0.12±0.05 | 3.40E-37 |
| HDAC8 | cg26399113 | 0.53±0.14 | 0.09±0.07 | 2.17E-73 | RPGR | cg07070940 | 0.4±0.1 | 0.16±0.09 | 4.48E-37 |
| OTUD5 | cg18256128 | 0.51±0.15 | 0.06±0.07 | 3.19E-73 | TCEAL2 | cg22543648 | 0.12±0.04 | 0.04±0.03 | 5.59E-37 |
| PCSK1N | cg06791102 | 0.57±0.17 | 0.06±0.08 | 4.29E-73 | FLJ11016 | cg02620228 | 0.42±0.08 | 0.24±0.07 | 5.83E-37 |
| BEX2 | cg11806565 | 0.4±0.12 | 0.04±0.06 | 8.22E-73 | GJB1 | cg16452396 | 0.64±0.06 | 0.78±0.05 | 6.85E-37 |
| HADH2 | cg03221436 | 0.39±0.11 | 0.06±0.05 | 8.29E-73 | CETN2 | cg08159444 | 0.4±0.1 | 0.17±0.09 | 1.16E-36 |
| LAMP2 | cg17552650 | 0.54±0.15 | 0.08±0.07 | 1.53E-72 | GK | cg04721883 | 0.18±0.06 | 0.07±0.04 | 1.62E-36 |
| FAM11A | cg20371650 | 0.41±0.12 | 0.06±0.05 | 1.69E-72 | TCEAL3 | cg21978299 | 0.34±0.1 | 0.16±0.07 | 6.10E-36 |
| PHF8 | cg07356189 | 0.47±0.13 | 0.08±0.06 | 3.23E-72 | DIAPH2 | cg16716983 | 0.17±0.06 | 0.07±0.03 | 9.34E-36 |
| CXorf17 | cg04533591 | 0.42±0.13 | 0.05±0.05 | 3.31E-72 | PHF6 | cg02132188 | 0.29±0.1 | 0.09±0.08 | 3.67E-35 |
| G6PD | cg11764747 | 0.42±0.12 | 0.08±0.05 | 4.89E-72 | ZNF75 | cg08214328 | 0.39±0.09 | 0.19±0.08 | 4.58E-35 |
| UTP14A | cg14625604 | 0.48±0.14 | 0.07±0.06 | 5.34E-72 | SMARCA1 | cg09202373 | 0.28±0.07 | 0.13±0.06 | 7.38E-35 |
| BCAP31 | cg01408383 | 0.58±0.16 | 0.09±0.07 | 5.88E-72 | KIAA1166 | cg10401803 | 0.23±0.07 | 0.08±0.06 | 1.18E-34 |
| PLP2 | cg17775283 | 0.52±0.14 | 0.1±0.06 | 6.84E-72 | SUHW3 | cg11291200 | 0.57±0.08 | 0.39±0.07 | 2.05E-34 |
| DXS9879E | cg24714666 | 0.44±0.12 | 0.07±0.06 | 7.07E-72 | SLC35A2 | cg22980351 | 0.24±0.07 | 0.1±0.05 | 2.36E-34 |
| BEX1 | cg21030483 | 0.41±0.12 | 0.05±0.05 | 7.80E-72 | FAM9C | cg14986136 | 0.8±0.08 | 0.92±0.04 | 2.39E-34 |
| HTATSF1 | cg09018810 | 0.65±0.19 | 0.08±0.09 | 8.43E-72 | FMR1 | cg06617418 | 0.46±0.13 | 0.22±0.08 | 3.73E-34 |
| RP1-112K5.2 | cg19696622 | 0.58±0.16 | 0.08±0.08 | 8.62E-72 | GDI1 | cg15329483 | 0.31±0.1 | 0.14±0.06 | 6.77E-34 |
| RPL36A | cg17843048 | 0.63±0.16 | 0.11±0.09 | 9.06E-72 | MECP2 | cg00600110 | 0.37±0.1 | 0.19±0.06 | 8.58E-34 |
| LOC389852 | cg25410053 | 0.33±0.1 | 0.05±0.04 | 9.87E-72 | RNF12 | cg11033833 | 0.28±0.08 | 0.12±0.06 | 1.40E-33 |
| CHST7 | cg04368919 | 0.71±0.19 | 0.1±0.1 | 1.17E-71 | CXorf56 | cg07911663 | 0.38±0.09 | 0.18±0.08 | 2.61E-33 |
| NKAP | cg18717447 | 0.4±0.11 | 0.06±0.05 | 1.20E-71 | CXorf41 | cg05813234 | 0.26±0.07 | 0.1±0.07 | 3.98E-33 |
| MAGEE1 | cg11653864 | 0.36±0.1 | 0.05±0.05 | 1.24E-71 | OGT | cg25531166 | 0.36±0.1 | 0.13±0.1 | 4.93E-33 |
| IRAK1 | cg00920960 | 0.6±0.16 | 0.09±0.08 | 1.51E-71 | SLC7A3 | cg19856594 | 0.33±0.08 | 0.15±0.08 | 8.10E-33 |
| TMLHE | cg06220755 | 0.34±0.1 | 0.05±0.04 | 1.59E-71 | RAB9P1 | cg23934633 | 0.76±0.05 | 0.63±0.06 | 8.68E-33 |
| FLJ30058 | cg24101388 | 0.38±0.11 | 0.07±0.04 | 1.69E-71 | BRWD3 | cg24352688 | 0.24±0.08 | 0.09±0.06 | 1.18E-32 |
| CNKSR2 | cg08693325 | 0.34±0.1 | 0.05±0.04 | 1.89E-71 | PIM2 | cg18044482 | 0.28±0.07 | 0.12±0.07 | 1.20E-32 |
| F8A1 | cg11810837 | 0.7±0.19 | 0.09±0.11 | 2.29E-71 | MAGEH1 | cg18389752 | 0.25±0.06 | 0.1±0.06 | 1.90E-32 |
| EBP | cg12919379 | 0.39±0.12 | 0.06±0.05 | 2.68E-71 | RP11-450P7.3 | cg21087701 | 0.18±0.06 | 0.07±0.04 | 2.96E-32 |
| NHS | cg17398312 | 0.29±0.09 | 0.05±0.04 | 2.79E-71 | KLF8 | cg06177698 | 0.21±0.07 | 0.07±0.06 | 6.29E-32 |
| ELK1 | cg11016745 | 0.58±0.15 | 0.11±0.08 | 2.87E-71 | RENBP | cg03445896 | 0.17±0.06 | 0.07±0.04 | 1.09E-31 |
| SYN1 | cg04735237 | 0.7±0.15 | 0.25±0.07 | 3.09E-71 | ABCB7 | cg15373633 | 0.28±0.07 | 0.12±0.07 | 1.12E-31 |
| RAB33A | cg23424962 | 0.58±0.14 | 0.13±0.08 | 3.49E-71 | MAGEA8 | cg05511752 | 0.68±0.06 | 0.82±0.07 | 1.88E-31 |
| TCEAL8 | cg19572242 | 0.42±0.11 | 0.06±0.06 | 6.38E-71 | KIAA1166 | cg23083672 | 0.21±0.06 | 0.07±0.06 | 2.43E-31 |
| AFF2 | cg13257485 | 0.41±0.12 | 0.06±0.05 | 7.26E-71 | ARMCX6 | cg04927982 | 0.22±0.09 | 0.06±0.07 | 1.62E-30 |
| DLG3 | cg23545272 | 0.44±0.12 | 0.08±0.06 | 7.68E-71 | GABRE | cg09261015 | 0.74±0.11 | 0.48±0.12 | 1.04E-29 |
| SOX3 | cg02165720 | 0.44±0.12 | 0.07±0.06 | 7.81E-71 | PAGE2 | cg26561773 | 0.79±0.06 | 0.92±0.06 | 1.10E-29 |
| CNKSR2 | cg25456959 | 0.47±0.14 | 0.06±0.07 | 1.44E-70 | MAGEB2 | cg10537079 | 0.75±0.08 | 0.91±0.07 | 1.29E-29 |
| PDK3 | cg17474651 | 0.54±0.13 | 0.14±0.07 | 1.50E-70 | NSBP1 | cg08434396 | 0.32±0.07 | 0.15±0.08 | 2.75E-29 |
| GPRASP2 | cg00280894 | 0.4±0.08 | 0.13±0.05 | 1.59E-70 | TMSB4X | cg20869203 | 0.31±0.09 | 0.14±0.08 | 3.00E-29 |
| MSL3L1 | cg25270201 | 0.42±0.11 | 0.11±0.05 | 2.11E-70 | EIF2S3 | cg06655100 | 0.12±0.05 | 0.05±0.03 | 3.83E-29 |
| ACSL4 | cg06340713 | 0.55±0.14 | 0.1±0.08 | 2.31E-70 | H2AFB3 | cg06057707 | 0.77±0.04 | 0.85±0.03 | 4.63E-29 |
| ATP7A | cg20622056 | 0.44±0.12 | 0.09±0.06 | 2.86E-70 | DACH2 | cg14708847 | 0.28±0.09 | 0.11±0.08 | 4.68E-29 |
| GK | cg14841098 | 0.48±0.14 | 0.07±0.07 | 3.02E-70 | ARHGAP4 | cg05206587 | 0.29±0.13 | 0.08±0.09 | 6.09E-29 |
| FAM50A | cg11944101 | 0.56±0.16 | 0.08±0.08 | 3.25E-70 | STK23 | cg23812886 | 0.67±0.11 | 0.38±0.14 | 6.46E-29 |
| XKRX | cg11049305 | 0.48±0.12 | 0.11±0.06 | 5.06E-70 | RBM3 | cg26572597 | 0.25±0.08 | 0.1±0.06 | 1.94E-28 |
| RPS6KA3 | cg10857345 | 0.32±0.09 | 0.04±0.04 | 5.56E-70 | SMS | cg17991347 | 0.28±0.07 | 0.14±0.07 | 2.46E-28 |
| RPL39 | cg21232685 | 0.37±0.11 | 0.06±0.05 | 6.52E-70 | UPF3B | cg14962776 | 0.29±0.09 | 0.14±0.06 | 2.55E-28 |
| AR | cg00662775 | 0.45±0.11 | 0.1±0.06 | 9.20E-70 | MAGEA3 | cg15636587 | 0.8±0.05 | 0.88±0.03 | 2.70E-28 |
| IRS4 | cg12517167 | 0.49±0.12 | 0.14±0.06 | 1.07E-69 | TDGF1 | cg19327844 | 0.51±0.07 | 0.64±0.06 | 2.85E-28 |
| SCML1 | cg17206029 | 0.5±0.13 | 0.09±0.07 | 1.48E-69 | PIN4 | cg20825323 | 0.26±0.09 | 0.12±0.06 | 4.92E-28 |
| CXorf42 | cg04544154 | 0.39±0.11 | 0.07±0.06 | 2.32E-69 | CSAG3A | cg22873668 | 0.81±0.05 | 0.89±0.03 | 8.29E-28 |
| CXorf26 | cg12434779 | 0.36±0.08 | 0.11±0.05 | 2.92E-69 | CXX1 | cg04493740 | 0.31±0.07 | 0.15±0.08 | 8.55E-28 |
| FLJ14503 | cg09176853 | 0.29±0.1 | 0.03±0.03 | 3.24E-69 | FLJ39827 | cg06532147 | 0.24±0.09 | 0.07±0.08 | 2.24E-27 |
| GPR173 | cg10818284 | 0.52±0.09 | 0.21±0.06 | 3.47E-69 | GAB3 | cg11174654 | 0.24±0.07 | 0.11±0.07 | 2.68E-27 |
| UXT | cg18564727 | 0.53±0.16 | 0.07±0.07 | 3.86E-69 | PCYT1B | cg21533271 | 0.26±0.08 | 0.12±0.07 | 5.35E-27 |
| TLE1 | cg01454134 | 0.47±0.12 | 0.09±0.07 | 6.49E-69 | XAGE3 | cg19845843 | 0.6±0.08 | 0.74±0.07 | 7.15E-27 |
| DNASE1L1 | cg18727700 | 0.55±0.16 | 0.08±0.08 | 6.70E-69 | ARD1A | cg25130333 | 0.39±0.08 | 0.2±0.1 | 7.43E-27 |
| MPP1 | cg21807589 | 0.41±0.11 | 0.07±0.06 | 7.22E-69 | SH3KBP1 | cg24038764 | 0.47±0.08 | 0.33±0.07 | 1.07E-26 |
| C1GALT1C1 | cg23279136 | 0.44±0.14 | 0.04±0.06 | 8.38E-69 | NKAP | cg02345317 | 0.36±0.09 | 0.19±0.08 | 1.11E-26 |
| MED12 | cg21090723 | 0.52±0.14 | 0.11±0.07 | 9.79E-69 | DKFZp564K142 | cg09607232 | 0.3±0.08 | 0.13±0.09 | 1.13E-26 |
| USP51 | cg24765005 | 0.35±0.11 | 0.04±0.05 | 1.90E-68 | MAGEA6 | cg06363801 | 0.81±0.04 | 0.88±0.03 | 1.29E-26 |
| PRAF2 | cg13837202 | 0.43±0.12 | 0.08±0.06 | 2.12E-68 | DKC1 | cg00862041 | 0.24±0.1 | 0.09±0.07 | 1.47E-26 |
| USP51 | cg18244029 | 0.39±0.1 | 0.08±0.05 | 2.84E-68 | CSAG1 | cg08300622 | 0.84±0.04 | 0.9±0.03 | 1.55E-26 |
| CDKL5 | cg01112452 | 0.3±0.09 | 0.05±0.04 | 3.15E-68 | CNGA2 | cg16144006 | 0.61±0.06 | 0.73±0.06 | 1.98E-26 |
| EFNB1 | cg04455999 | 0.49±0.12 | 0.12±0.07 | 3.26E-68 | XPNPEP2 | cg02678356 | 0.47±0.09 | 0.27±0.11 | 2.40E-26 |
| AMMECR1 | cg11822772 | 0.42±0.13 | 0.06±0.06 | 3.29E-68 | KLF8 | cg05935584 | 0.2±0.07 | 0.09±0.05 | 5.02E-26 |
| MAOA | cg14520892 | 0.51±0.13 | 0.09±0.08 | 3.48E-68 | SLITRK2 | cg02264284 | 0.26±0.06 | 0.11±0.08 | 5.97E-26 |
| EFNB1 | cg23378094 | 0.49±0.14 | 0.08±0.07 | 3.65E-68 | MID1 | cg19441691 | 0.21±0.04 | 0.12±0.05 | 1.58E-25 |
| ARHGEF9 | cg10536534 | 0.5±0.14 | 0.08±0.07 | 3.72E-68 | AR | cg20154346 | 0.2±0.06 | 0.08±0.06 | 2.83E-25 |
| CXorf40B | cg02205962 | 0.46±0.12 | 0.11±0.06 | 3.85E-68 | RRAGB | cg12431196 | 0.17±0.05 | 0.06±0.06 | 7.48E-25 |
| ARMCX2 | cg18468467 | 0.49±0.13 | 0.08±0.07 | 4.13E-68 | UPF3B | cg26966384 | 0.28±0.11 | 0.12±0.07 | 8.93E-25 |
| ATP6AP1 | cg18239702 | 0.51±0.15 | 0.08±0.07 | 5.05E-68 | RBBP7 | cg14168975 | 0.42±0.06 | 0.3±0.06 | 1.59E-24 |
| TSC22D3 | cg06899582 | 0.43±0.13 | 0.05±0.07 | 5.10E-68 | MTM1 | cg26547788 | 0.76±0.04 | 0.65±0.06 | 3.16E-24 |
| UBL4A | cg10591659 | 0.55±0.16 | 0.09±0.08 | 5.41E-68 | RP3-473B4.1 | cg15720064 | 0.22±0.09 | 0.08±0.07 | 4.24E-24 |
| ESX1 | cg08695223 | 0.29±0.09 | 0.05±0.04 | 6.31E-68 | INGX | cg04929865 | 0.39±0.1 | 0.16±0.13 | 4.32E-24 |
| SUV39H1 | cg21966410 | 0.42±0.12 | 0.09±0.05 | 8.42E-68 | IL1RAPL2 | cg22762309 | 0.14±0.04 | 0.07±0.04 | 7.36E-24 |
| SLC9A7 | cg18364820 | 0.56±0.15 | 0.1±0.09 | 9.66E-68 | NR0B1 | cg21516819 | 0.34±0.07 | 0.2±0.07 | 2.05E-23 |
| ZNF673 | cg13758677 | 0.38±0.11 | 0.05±0.05 | 1.24E-67 | UBQLN2 | cg09144786 | 0.3±0.08 | 0.17±0.07 | 2.20E-23 |
| TBX22 | cg14719055 | 0.31±0.09 | 0.06±0.04 | 1.30E-67 | SAT | cg19481953 | 0.16±0.05 | 0.07±0.05 | 3.04E-23 |
| FAM58A | cg00690893 | 0.42±0.12 | 0.07±0.06 | 1.82E-67 | LRRC2 | cg17074212 | 0.75±0.05 | 0.82±0.04 | 3.27E-23 |
| RPL39 | cg03959079 | 0.45±0.13 | 0.08±0.06 | 2.21E-67 | SLC7A3 | cg10009003 | 0.17±0.06 | 0.07±0.06 | 3.44E-23 |
| STARD8 | cg20065832 | 0.34±0.09 | 0.1±0.04 | 5.50E-67 | SLITRK4 | cg12384303 | 0.32±0.08 | 0.16±0.09 | 4.16E-23 |
| FHL1 | cg13915726 | 0.38±0.1 | 0.09±0.05 | 5.89E-67 | RBM10 | cg20842040 | 0.66±0.1 | 0.47±0.1 | 4.28E-23 |
| LMO6 | cg06657741 | 0.32±0.09 | 0.07±0.04 | 6.78E-67 | DLG3 | cg19206010 | 0.22±0.06 | 0.11±0.06 | 6.47E-23 |
| CCDC22 | cg25758314 | 0.41±0.11 | 0.07±0.06 | 7.27E-67 | GPR173 | cg09665351 | 0.23±0.06 | 0.13±0.06 | 8.63E-23 |
| PLXNA3 | cg06822229 | 0.53±0.15 | 0.1±0.07 | 1.17E-66 | POU3F4 | cg23079782 | 0.26±0.06 | 0.14±0.07 | 1.04E-22 |
| F8A1 | cg11353032 | 0.64±0.17 | 0.11±0.11 | 1.23E-66 | GSPT2 | cg21509846 | 0.34±0.07 | 0.16±0.1 | 1.67E-22 |
| EFNB1 | cg10321196 | 0.45±0.11 | 0.1±0.06 | 1.75E-66 | FLJ31204 | cg12382941 | 0.26±0.07 | 0.12±0.08 | 1.77E-22 |
| FLJ10178 | cg02326006 | 0.39±0.11 | 0.06±0.06 | 1.87E-66 | TSPAN7 | cg19618706 | 0.24±0.05 | 0.16±0.04 | 2.99E-22 |
| ELF4 | cg05921207 | 0.45±0.13 | 0.06±0.07 | 2.20E-66 | NLGN3 | cg03142203 | 0.52±0.12 | 0.29±0.14 | 3.12E-22 |
| PHF16 | cg17032587 | 0.53±0.14 | 0.12±0.07 | 2.92E-66 | MAGEA2 | cg00981643 | 0.81±0.05 | 0.87±0.03 | 3.25E-22 |
| GPC4 | cg08093211 | 0.46±0.15 | 0.04±0.08 | 3.24E-66 | PRPS1 | cg20473453 | 0.22±0.06 | 0.1±0.07 | 4.87E-22 |
| NHS | cg13792569 | 0.4±0.12 | 0.05±0.06 | 4.50E-66 | EFNB1 | cg12330929 | 0.22±0.06 | 0.12±0.06 | 5.83E-22 |
| MOSPD2 | cg10926623 | 0.42±0.11 | 0.09±0.06 | 5.59E-66 | ATG4A | cg17239363 | 0.38±0.09 | 0.24±0.07 | 6.61E-22 |
| KIF4A | cg15409266 | 0.39±0.11 | 0.09±0.05 | 5.63E-66 | PFC | cg16561743 | 0.53±0.11 | 0.33±0.12 | 8.50E-22 |
| AR | cg04544498 | 0.53±0.15 | 0.1±0.08 | 6.86E-66 | HDAC6 | cg23926715 | 0.23±0.08 | 0.11±0.07 | 1.57E-21 |
| DYNLT3 | cg24636657 | 0.37±0.1 | 0.07±0.06 | 1.22E-65 | FLJ43276 | cg19740375 | 0.06±0.04 | 0.14±0.04 | 1.82E-21 |
| NDUFB11 | cg01375994 | 0.39±0.11 | 0.07±0.06 | 1.29E-65 | ZNF185 | cg26222407 | 0.59±0.08 | 0.43±0.1 | 2.57E-21 |
| MSN | cg15309236 | 0.32±0.1 | 0.07±0.04 | 1.31E-65 | ARR3 | cg04924696 | 0.7±0.06 | 0.81±0.07 | 2.94E-21 |
| ABCB7 | cg22705954 | 0.28±0.08 | 0.06±0.04 | 1.88E-65 | YIPF6 | cg23232084 | 0.52±0.1 | 0.28±0.15 | 3.52E-21 |
| WDR45 | cg16499669 | 0.54±0.15 | 0.11±0.08 | 2.71E-65 | FLJ43276 | cg22084336 | 0.07±0.04 | 0.14±0.04 | 3.63E-21 |
| VBP1 | cg00128197 | 0.55±0.16 | 0.09±0.09 | 4.26E-65 | ATP1B4 | cg18780401 | 0.69±0.09 | 0.82±0.07 | 1.00E-20 |
| ATP6AP2 | cg06306751 | 0.49±0.14 | 0.08±0.08 | 4.70E-65 | SLC10A3 | cg22858728 | 0.78±0.04 | 0.68±0.06 | 2.24E-20 |
| CETN2 | cg25813820 | 0.53±0.15 | 0.06±0.09 | 5.32E-65 | ARHGAP4 | cg06325687 | 0.51±0.09 | 0.31±0.13 | 2.37E-20 |
| STAG2 | cg15375586 | 0.41±0.11 | 0.09±0.06 | 5.56E-65 | ASB12 | cg24774956 | 0.42±0.08 | 0.58±0.1 | 3.24E-20 |
| RAB33A | cg06731599 | 0.49±0.14 | 0.08±0.08 | 6.31E-65 | MAOB | cg26776077 | 0.22±0.05 | 0.13±0.06 | 3.81E-20 |
| MTMR1 | cg00618396 | 0.45±0.13 | 0.07±0.07 | 7.05E-65 | TAF1 | cg23163644 | 0.4±0.1 | 0.23±0.11 | 5.83E-20 |
| SLC25A14 | cg03791917 | 0.49±0.12 | 0.12±0.07 | 7.31E-65 | PGRMC1 | cg04875162 | 0.8±0.05 | 0.88±0.05 | 8.98E-20 |
| MAOA | cg18731813 | 0.35±0.1 | 0.06±0.05 | 7.41E-65 | CXorf12 | cg08750326 | 0.16±0.05 | 0.09±0.04 | 1.06E-19 |
| ARAF | cg09581098 | 0.36±0.12 | 0.05±0.05 | 7.48E-65 | GPR101 | cg10349665 | 0.24±0.07 | 0.12±0.07 | 1.26E-19 |
| PCYT1B | cg20244073 | 0.45±0.11 | 0.13±0.07 | 7.89E-65 | PIR | cg18511445 | 0.1±0.03 | 0.05±0.03 | 1.50E-19 |
| PDCD8 | cg20430101 | 0.44±0.12 | 0.09±0.06 | 8.27E-65 | MAGED1 | cg17196716 | 0.21±0.05 | 0.12±0.06 | 1.79E-19 |
| ARMCX1 | cg18233735 | 0.42±0.11 | 0.11±0.06 | 1.25E-64 | UTP14A | cg15521097 | 0.26±0.07 | 0.14±0.08 | 3.14E-19 |
| FTSJ1 | cg05961595 | 0.64±0.16 | 0.13±0.1 | 1.30E-64 | COVA1 | cg09229960 | 0.18±0.06 | 0.09±0.06 | 3.69E-19 |
| EFNB1 | cg15014034 | 0.3±0.1 | 0.05±0.04 | 1.84E-64 | TCEAL3 | cg23986186 | 0.24±0.07 | 0.13±0.07 | 4.27E-19 |
| MID2 | cg19740287 | 0.55±0.14 | 0.12±0.09 | 5.52E-64 | MAGEE2 | cg19963797 | 0.24±0.06 | 0.14±0.06 | 4.96E-19 |
| EFNB1 | cg22899145 | 0.53±0.15 | 0.11±0.08 | 6.76E-64 | ZDHHC15 | cg04765675 | 0.33±0.07 | 0.2±0.09 | 5.76E-19 |
| SYTL4 | cg11640565 | 0.31±0.1 | 0.05±0.04 | 7.12E-64 | ARAF | cg03291145 | 0.21±0.06 | 0.1±0.07 | 5.82E-19 |
| HMGB3 | cg18049750 | 0.37±0.1 | 0.09±0.05 | 7.40E-64 | AR | cg15977272 | 0.45±0.11 | 0.21±0.17 | 6.33E-19 |
| MSN | cg08965337 | 0.6±0.13 | 0.22±0.07 | 9.30E-64 | PSMD10 | cg17189778 | 0.19±0.05 | 0.1±0.05 | 7.55E-19 |
| NKRF | cg21515329 | 0.43±0.13 | 0.09±0.06 | 1.68E-63 | CHRDL1 | cg11681617 | 0.16±0.05 | 0.07±0.05 | 8.28E-19 |
| SYN1 | cg13174077 | 0.42±0.13 | 0.06±0.07 | 1.87E-63 | MXRA5 | cg12485020 | 0.42±0.08 | 0.54±0.07 | 8.99E-19 |
| MED12 | cg04920616 | 0.44±0.13 | 0.09±0.06 | 2.62E-63 | FAM9C | cg13226591 | 0.77±0.05 | 0.85±0.05 | 9.52E-19 |
| CD99L2 | cg24428913 | 0.39±0.12 | 0.07±0.06 | 3.44E-63 | OTC | cg05801573 | 0.61±0.08 | 0.76±0.1 | 1.02E-18 |
| MLLT7 | cg23921534 | 0.21±0.07 | 0.03±0.03 | 3.94E-63 | ITGB1BP2 | cg11648996 | 0.48±0.07 | 0.33±0.11 | 1.64E-18 |
| SLC16A2 | cg22497867 | 0.32±0.08 | 0.09±0.05 | 4.87E-63 | CXorf56 | cg21836062 | 0.47±0.06 | 0.36±0.07 | 1.67E-18 |
| FLJ25444 | cg04499381 | 0.38±0.1 | 0.09±0.06 | 5.21E-63 | HPRT1 | cg01592593 | 0.28±0.07 | 0.15±0.09 | 2.09E-18 |
| ARHGAP6 | cg11661234 | 0.32±0.09 | 0.06±0.05 | 5.32E-63 | FRMPD4 | cg06620254 | 0.11±0.04 | 0.06±0.03 | 3.94E-18 |
| PHKA1 | cg11272332 | 0.41±0.11 | 0.08±0.07 | 7.03E-63 | SH3BGRL | cg26328611 | 0.24±0.05 | 0.15±0.06 | 8.27E-18 |
| APEX2 | cg26944151 | 0.48±0.13 | 0.1±0.08 | 7.34E-63 | NOX1 | cg26412379 | 0.36±0.07 | 0.26±0.06 | 1.17E-17 |
| PIN4 | cg20648827 | 0.34±0.11 | 0.07±0.04 | 7.52E-63 | SRPX | cg07824317 | 0.43±0.09 | 0.32±0.07 | 1.76E-17 |
| RBM3 | cg11598929 | 0.45±0.11 | 0.12±0.07 | 9.39E-63 | TLR8 | cg06944050 | 0.53±0.07 | 0.36±0.12 | 1.80E-17 |
| RPL10 | cg18414950 | 0.54±0.16 | 0.08±0.09 | 2.06E-62 | PNMA3 | cg04872051 | 0.61±0.09 | 0.42±0.14 | 2.00E-17 |
| SNX12 | cg14755341 | 0.22±0.07 | 0.04±0.03 | 2.27E-62 | FLJ31204 | cg10864286 | 0.37±0.07 | 0.26±0.07 | 2.70E-17 |
| TFE3 | cg01228667 | 0.43±0.12 | 0.12±0.05 | 2.46E-62 | WAS | cg23709838 | 0.64±0.1 | 0.45±0.14 | 3.87E-17 |
| NDUFB11 | cg13088755 | 0.55±0.13 | 0.19±0.07 | 6.60E-62 | SAGE1 | cg19055639 | 0.69±0.05 | 0.76±0.05 | 4.23E-17 |
| RP13-360B22.2 | cg11331769 | 0.4±0.12 | 0.09±0.06 | 1.18E-61 | ARMCX3 | cg11964613 | 0.24±0.07 | 0.14±0.06 | 7.21E-17 |
| LONRF3 | cg06494770 | 0.37±0.11 | 0.08±0.06 | 2.36E-61 | TMLHE | cg06691299 | 0.27±0.06 | 0.16±0.08 | 1.37E-16 |
| GLUD2 | cg10455133 | 0.2±0.06 | 0.04±0.03 | 2.53E-61 | MAGEH1 | cg00691822 | 0.2±0.07 | 0.08±0.08 | 2.18E-16 |
| RP11-311P8.3 | cg18449462 | 0.45±0.13 | 0.11±0.07 | 4.50E-61 | ZNF185 | cg15200096 | 0.67±0.1 | 0.5±0.13 | 3.16E-16 |
| GABRQ | cg11233153 | 0.4±0.11 | 0.11±0.06 | 4.99E-61 | LW-1 | cg23776892 | 0.84±0.03 | 0.88±0.03 | 3.19E-16 |
| FLJ39827 | cg18665563 | 0.32±0.11 | 0.05±0.05 | 5.34E-61 | CNGA2 | cg04238548 | 0.41±0.07 | 0.54±0.1 | 3.31E-16 |
| EGFL6 | cg00415494 | 0.29±0.09 | 0.06±0.04 | 1.69E-60 | MID1IP1 | cg25226891 | 0.27±0.09 | 0.15±0.08 | 3.58E-16 |
| TMEM47 | cg20832009 | 0.33±0.08 | 0.09±0.05 | 1.91E-60 | ELF4 | cg03992069 | 0.77±0.12 | 0.51±0.2 | 4.14E-16 |
| SH3KBP1 | cg25317260 | 0.43±0.12 | 0.1±0.07 | 2.18E-60 | GAB3 | cg12468189 | 0.21±0.07 | 0.1±0.07 | 4.20E-16 |
| BHLHB9 | cg14674582 | 0.39±0.1 | 0.11±0.06 | 2.85E-60 | CXorf9 | cg17231524 | 0.7±0.09 | 0.49±0.16 | 4.23E-16 |
| ATRX | cg01361446 | 0.57±0.15 | 0.12±0.1 | 3.82E-60 | CCDC22 | cg17718322 | 0.71±0.1 | 0.55±0.12 | 5.31E-16 |
| WDR13 | cg24286301 | 0.57±0.14 | 0.19±0.08 | 4.75E-60 | MORF4L2 | cg25488547 | 0.17±0.07 | 0.08±0.06 | 5.35E-16 |
| BCORL1 | cg22617367 | 0.35±0.11 | 0.07±0.05 | 5.62E-60 | TSPAN6 | cg05920720 | 0.21±0.05 | 0.12±0.07 | 8.40E-16 |
| CXX1 | cg26414720 | 0.38±0.11 | 0.08±0.06 | 6.64E-60 | MAGEA3 | cg06904813 | 0.85±0.04 | 0.9±0.03 | 8.49E-16 |
| ARMCX5 | cg11291009 | 0.27±0.08 | 0.06±0.04 | 7.01E-60 | SYTL4 | cg00536175 | 0.22±0.05 | 0.14±0.06 | 9.50E-16 |
| GNL3L | cg09324116 | 0.44±0.12 | 0.11±0.07 | 8.11E-60 | RRAGB | cg13771629 | 0.17±0.06 | 0.08±0.07 | 2.52E-15 |
| TSPAN7 | cg21046413 | 0.31±0.09 | 0.08±0.05 | 8.21E-60 | PLAC1 | cg14107638 | 0.52±0.07 | 0.63±0.09 | 3.69E-15 |
| PRPS2 | cg20085077 | 0.4±0.12 | 0.08±0.07 | 1.29E-59 | YIPF6 | cg14506668 | 0.25±0.06 | 0.16±0.07 | 4.17E-15 |
| CD99L2 | cg15480941 | 0.43±0.13 | 0.07±0.08 | 1.29E-59 | DUSP9 | cg13201322 | 0.34±0.08 | 0.22±0.08 | 5.98E-15 |
| MGC39606 | cg08769044 | 0.44±0.12 | 0.11±0.07 | 1.33E-59 | CXorf42 | cg23269489 | 0.26±0.06 | 0.15±0.08 | 7.91E-15 |
| IDH3G | cg26545968 | 0.47±0.12 | 0.12±0.08 | 1.38E-59 | CHM | cg24779040 | 0.68±0.13 | 0.51±0.13 | 2.38E-14 |
| LAMP2 | cg14356114 | 0.41±0.12 | 0.08±0.07 | 1.53E-59 | ITIH5L | cg14784348 | 0.45±0.07 | 0.58±0.11 | 3.77E-14 |
| GPRASP2 | cg22148243 | 0.37±0.08 | 0.13±0.06 | 2.46E-59 | GUCY2F | cg23407366 | 0.56±0.07 | 0.69±0.11 | 3.88E-14 |
| TCEAL1 | cg19062189 | 0.41±0.1 | 0.1±0.07 | 2.54E-59 | RPL10 | cg02690554 | 0.25±0.09 | 0.15±0.08 | 4.28E-14 |
| PSMD10 | cg17205788 | 0.34±0.1 | 0.08±0.05 | 2.90E-59 | AVPR2 | cg16227775 | 0.79±0.07 | 0.68±0.09 | 4.36E-14 |
| WWC3 | cg11505080 | 0.33±0.11 | 0.05±0.05 | 3.03E-59 | PNPLA4 | cg04282422 | 0.32±0.07 | 0.24±0.07 | 6.87E-14 |
| ZMYM3 | cg03127543 | 0.35±0.11 | 0.05±0.06 | 4.78E-59 | DKC1 | cg11325578 | 0.29±0.07 | 0.19±0.08 | 8.68E-14 |
| FUNDC2 | cg25376316 | 0.41±0.11 | 0.12±0.06 | 5.21E-59 | BMP15 | cg26116400 | 0.46±0.06 | 0.61±0.13 | 1.18E-13 |
| ZNF449 | cg18653991 | 0.4±0.11 | 0.07±0.07 | 5.33E-59 | WBP5 | cg14600040 | 0.29±0.08 | 0.17±0.1 | 2.49E-13 |
| WDR44 | cg12799835 | 0.4±0.1 | 0.08±0.08 | 5.71E-59 | PAGE4 | cg04241572 | 0.85±0.04 | 0.89±0.03 | 2.95E-13 |
| PHF16 | cg18535534 | 0.51±0.15 | 0.11±0.09 | 6.60E-59 | POU3F4 | cg19388557 | 0.16±0.05 | 0.09±0.05 | 4.00E-13 |
| AR | cg06959635 | 0.27±0.08 | 0.07±0.04 | 6.92E-59 | DMRTC1 | cg26606552 | 0.8±0.03 | 0.84±0.03 | 4.57E-13 |
| FLJ25444 | cg10339201 | 0.42±0.12 | 0.12±0.06 | 6.92E-59 | SSX4 | cg09917461 | 0.74±0.05 | 0.81±0.06 | 5.61E-13 |
| IRAK1 | cg18780751 | 0.34±0.1 | 0.07±0.06 | 7.20E-59 | RBM10 | cg19585882 | 0.6±0.11 | 0.47±0.11 | 1.38E-12 |
| ZNF449 | cg01257202 | 0.4±0.11 | 0.1±0.06 | 8.36E-59 | DPPA3 | cg04405541 | 0.52±0.08 | 0.42±0.08 | 1.43E-12 |
| MID2 | cg19002579 | 0.35±0.09 | 0.09±0.06 | 1.42E-58 | ATP11C | cg17693270 | 0.46±0.06 | 0.38±0.06 | 1.50E-12 |
| EMD | cg03575468 | 0.52±0.13 | 0.17±0.07 | 1.49E-58 | TLR7 | cg14642832 | 0.7±0.12 | 0.55±0.13 | 1.53E-12 |
| CHRDL1 | cg05254049 | 0.3±0.09 | 0.07±0.05 | 1.67E-58 | OGT | cg03704031 | 0.1±0.04 | 0.06±0.04 | 1.81E-12 |
| GPC4 | cg14063673 | 0.29±0.08 | 0.07±0.04 | 1.88E-58 | CLCN5 | cg18616655 | 0.61±0.07 | 0.72±0.1 | 2.05E-12 |
| MECP2 | cg10783042 | 0.26±0.08 | 0.05±0.04 | 2.05E-58 | MAGEC3 | cg02148711 | 0.62±0.05 | 0.69±0.06 | 2.26E-12 |
| HTATSF1 | cg19138060 | 0.37±0.11 | 0.06±0.07 | 2.26E-58 | TCEAL4 | cg00141845 | 0.31±0.09 | 0.21±0.08 | 2.26E-12 |
| UBQLN2 | cg01872771 | 0.41±0.11 | 0.12±0.06 | 3.29E-58 | FMR1NB | cg21462299 | 0.79±0.06 | 0.85±0.04 | 2.30E-12 |
| TRO | cg06457357 | 0.31±0.1 | 0.05±0.06 | 3.68E-58 | LOC389852 | cg15201909 | 0.55±0.04 | 0.5±0.04 | 2.33E-12 |
| CSTF2 | cg18091964 | 0.25±0.07 | 0.06±0.04 | 4.26E-58 | PCTK1 | cg06273903 | 0.13±0.05 | 0.07±0.05 | 2.35E-12 |
| LDOC1 | cg13991029 | 0.42±0.09 | 0.15±0.06 | 4.93E-58 | FRMPD4 | cg16390856 | 0.17±0.06 | 0.08±0.08 | 2.82E-12 |
| CDKL5 | cg14186071 | 0.32±0.1 | 0.06±0.05 | 7.22E-58 | MAGEC2 | cg14815778 | 0.81±0.05 | 0.86±0.03 | 3.33E-12 |
| FAM3A | cg25086957 | 0.28±0.09 | 0.06±0.04 | 7.63E-58 | DIAPH2 | cg07238167 | 0.08±0.03 | 0.05±0.02 | 4.85E-12 |
| BEX2 | cg24034992 | 0.43±0.12 | 0.11±0.07 | 8.73E-58 | AVPR2 | cg18624866 | 0.44±0.07 | 0.35±0.07 | 5.50E-12 |
| DACH2 | cg00977690 | 0.48±0.11 | 0.13±0.09 | 1.15E-57 | CXorf38 | cg17571782 | 0.14±0.1 | 0.07±0.04 | 7.05E-12 |
| PRDX4 | cg07489003 | 0.35±0.1 | 0.1±0.06 | 1.48E-57 | CDX4 | cg19964192 | 0.34±0.07 | 0.27±0.06 | 8.07E-12 |
| MLLT7 | cg08559914 | 0.24±0.09 | 0.04±0.03 | 1.58E-57 | LAS1L | cg00497084 | 0.19±0.05 | 0.12±0.06 | 9.08E-12 |
| AR | cg11839979 | 0.3±0.09 | 0.06±0.05 | 2.13E-57 | HDAC6 | cg09950034 | 0.31±0.1 | 0.21±0.08 | 1.65E-11 |
| TSC22D3 | cg27627570 | 0.26±0.08 | 0.06±0.04 | 2.33E-57 | INGX | cg02847500 | 0.27±0.08 | 0.14±0.13 | 3.53E-11 |
| ZDHHC15 | cg10530733 | 0.35±0.1 | 0.06±0.06 | 2.66E-57 | LHFPL1 | cg20119635 | 0.28±0.05 | 0.2±0.07 | 4.04E-11 |
| IL1RAPL1 | cg26035633 | 0.43±0.09 | 0.14±0.07 | 2.79E-57 | FLJ11016 | cg04755662 | 0.14±0.05 | 0.08±0.06 | 4.26E-11 |
| RP3-473B4.1 | cg00874863 | 0.34±0.1 | 0.09±0.05 | 5.19E-57 | TIMM8A | cg25670900 | 0.34±0.05 | 0.26±0.07 | 4.68E-11 |
| IDH3G | cg25359581 | 0.26±0.08 | 0.06±0.04 | 6.02E-57 | PABPC5 | cg04394101 | 0.15±0.06 | 0.09±0.04 | 5.74E-11 |
| TIMM8A | cg17542495 | 0.3±0.08 | 0.07±0.05 | 6.89E-57 | KLHL4 | cg16082125 | 0.33±0.05 | 0.28±0.05 | 5.99E-11 |
| BHLHB9 | cg00582372 | 0.28±0.08 | 0.09±0.04 | 8.33E-57 | GPR143 | cg10560144 | 0.2±0.07 | 0.14±0.05 | 6.72E-11 |
| PGK1 | cg26964989 | 0.42±0.11 | 0.11±0.07 | 2.05E-56 | RENBP | cg22745747 | 0.86±0.08 | 0.73±0.12 | 6.89E-11 |
| MAGED4 | cg22189618 | 0.27±0.08 | 0.06±0.05 | 2.39E-56 | CAPN6 | cg18869368 | 0.24±0.06 | 0.35±0.11 | 7.40E-11 |
| SNX12 | cg14368286 | 0.37±0.11 | 0.08±0.07 | 2.88E-56 | WBP5 | cg14457691 | 0.2±0.06 | 0.13±0.06 | 7.46E-11 |
| MOSPD2 | cg18930892 | 0.24±0.08 | 0.05±0.04 | 6.77E-56 | CD40LG | cg11479591 | 0.69±0.09 | 0.57±0.11 | 8.92E-11 |
| MASK | cg21290885 | 0.31±0.09 | 0.08±0.05 | 7.94E-56 | KLHL4 | cg07147350 | 0.17±0.05 | 0.11±0.06 | 9.25E-11 |
| BRWD3 | cg02575859 | 0.31±0.1 | 0.08±0.04 | 9.74E-56 | FLJ20582 | cg15798455 | 0.57±0.11 | 0.44±0.11 | 9.48E-11 |
| SCML1 | cg27286999 | 0.46±0.12 | 0.12±0.09 | 1.13E-55 | TRPC5 | cg12391921 | 0.47±0.08 | 0.56±0.09 | 1.61E-10 |
| UBE2A | cg05655041 | 0.27±0.1 | 0.05±0.04 | 1.20E-55 | ARMCX2 | cg21395967 | 0.23±0.06 | 0.14±0.09 | 1.84E-10 |
| VBP1 | cg11609760 | 0.3±0.09 | 0.09±0.05 | 1.85E-55 | TCEAL7 | cg13723482 | 0.74±0.05 | 0.79±0.05 | 2.02E-10 |
| CXorf53 | cg25268283 | 0.47±0.11 | 0.17±0.07 | 2.11E-55 | RIBC1 | cg12900739 | 0.11±0.05 | 0.06±0.04 | 2.13E-10 |
| BIRC4 | cg09094355 | 0.47±0.09 | 0.18±0.08 | 2.57E-55 | KCND1 | cg06051662 | 0.19±0.06 | 0.12±0.07 | 2.35E-10 |
| CSTF2 | cg07374632 | 0.24±0.07 | 0.06±0.04 | 2.91E-55 | MTM1 | cg26981881 | 0.38±0.08 | 0.3±0.09 | 2.58E-10 |
| PLXNA3 | cg21067846 | 0.4±0.11 | 0.11±0.07 | 3.50E-55 | CLIC2 | cg20739508 | 0.29±0.08 | 0.2±0.09 | 2.63E-10 |
| EFNB1 | cg11236244 | 0.4±0.12 | 0.11±0.07 | 3.55E-55 | BIRC4 | cg00404599 | 0.24±0.06 | 0.17±0.08 | 3.49E-10 |
| NONO | cg26309951 | 0.35±0.09 | 0.11±0.06 | 3.57E-55 | TAZ | cg10575735 | 0.64±0.11 | 0.52±0.12 | 3.74E-10 |
| IGSF1 | cg04397441 | 0.35±0.1 | 0.09±0.06 | 3.83E-55 | GSPT2 | cg20816612 | 0.57±0.08 | 0.71±0.15 | 5.14E-10 |
| SCML2 | cg07581973 | 0.42±0.12 | 0.09±0.08 | 6.69E-55 | MAGEA12 | cg01885202 | 0.71±0.1 | 0.59±0.12 | 6.36E-10 |
| IRS4 | cg07688234 | 0.31±0.09 | 0.08±0.05 | 7.81E-55 | NXF2 | cg12732953 | 0.72±0.06 | 0.79±0.07 | 9.39E-10 |
| ACSL4 | cg14807303 | 0.45±0.12 | 0.15±0.07 | 9.30E-55 | PAGE2 | cg05376895 | 0.81±0.05 | 0.87±0.07 | 1.10E-09 |
| MTMR8 | cg08785133 | 0.42±0.1 | 0.13±0.08 | 1.14E-54 | EIF2S3 | cg26681123 | 0.22±0.05 | 0.28±0.06 | 1.10E-09 |
| GRIA3 | cg22835805 | 0.4±0.13 | 0.08±0.07 | 1.55E-54 | SYTL5 | cg12251508 | 0.61±0.05 | 0.68±0.08 | 2.73E-09 |
| ZCCHC12 | cg11371160 | 0.34±0.1 | 0.08±0.07 | 2.39E-54 | SLC6A8 | cg06834983 | 0.75±0.09 | 0.58±0.2 | 3.16E-09 |
| KCNE1L | cg04882894 | 0.63±0.08 | 0.84±0.05 | 3.41E-54 | CHST7 | cg27024922 | 0.45±0.08 | 0.33±0.13 | 3.82E-09 |
| NONO | cg06162422 | 0.42±0.11 | 0.11±0.08 | 7.08E-54 | WDR40B | cg21934269 | 0.37±0.06 | 0.29±0.09 | 5.50E-09 |
| SLC35A2 | cg13621440 | 0.36±0.13 | 0.07±0.06 | 7.46E-54 | NXF2 | cg27490193 | 0.59±0.09 | 0.7±0.12 | 5.55E-09 |
| PDZD4 | cg17354708 | 0.38±0.12 | 0.08±0.07 | 1.01E-53 | CXorf6 | cg15664701 | 0.48±0.09 | 0.61±0.15 | 7.74E-09 |
| XK | cg08248532 | 0.32±0.09 | 0.1±0.05 | 1.80E-53 | GLT28D1 | cg22759686 | 0.63±0.12 | 0.5±0.14 | 7.90E-09 |
| PRPS2 | cg07137581 | 0.3±0.09 | 0.1±0.04 | 1.82E-53 | DMD | cg21183872 | 0.55±0.12 | 0.43±0.13 | 8.54E-09 |
| NR0B1 | cg08843314 | 0.31±0.09 | 0.09±0.05 | 2.38E-53 | RNF113A | cg22562335 | 0.65±0.13 | 0.52±0.14 | 8.68E-09 |
| FLNA | cg06313930 | 0.45±0.09 | 0.19±0.07 | 2.51E-53 | FLJ20298 | cg20855303 | 0.14±0.07 | 0.09±0.05 | 1.29E-08 |
| PHKA1 | cg20062122 | 0.45±0.11 | 0.15±0.08 | 5.38E-53 | UBL4A | cg22601215 | 0.81±0.09 | 0.7±0.13 | 1.43E-08 |
| WDR44 | cg08496601 | 0.37±0.11 | 0.09±0.07 | 5.52E-53 | SSX5 | cg23066860 | 0.6±0.1 | 0.71±0.13 | 1.62E-08 |
| AFF2 | cg26418544 | 0.3±0.08 | 0.08±0.06 | 8.33E-53 | CXorf12 | cg06338119 | 0.14±0.05 | 0.09±0.06 | 1.64E-08 |
| RAB39B | cg01718602 | 0.28±0.09 | 0.08±0.05 | 9.61E-53 | HCCS | cg23947872 | 0.36±0.07 | 0.29±0.07 | 2.49E-08 |
| PORCN | cg02096520 | 0.4±0.08 | 0.18±0.06 | 9.81E-53 | NXF5 | cg15008991 | 0.78±0.07 | 0.7±0.09 | 2.79E-08 |
| SH3BGRL | cg11854877 | 0.39±0.1 | 0.09±0.08 | 1.29E-52 | IL2RG | cg14570389 | 0.43±0.06 | 0.36±0.07 | 3.29E-08 |
| FMR1 | cg07780118 | 0.42±0.12 | 0.12±0.07 | 1.29E-52 | ACTRT1 | cg07242375 | 0.62±0.06 | 0.69±0.08 | 3.66E-08 |
| FAM50A | cg11565248 | 0.37±0.11 | 0.11±0.06 | 1.82E-52 | PNMA6A | cg06416685 | 0.68±0.08 | 0.58±0.12 | 5.02E-08 |
| RBMX2 | cg25893483 | 0.42±0.12 | 0.11±0.08 | 2.14E-52 | PLXNB3 | cg01632517 | 0.75±0.11 | 0.63±0.14 | 5.05E-08 |
| PQBP1 | cg22731373 | 0.26±0.07 | 0.07±0.05 | 2.46E-52 | P2RY4 | cg25708912 | 0.63±0.08 | 0.71±0.1 | 5.13E-08 |
| CASK | cg15454483 | 0.44±0.12 | 0.11±0.08 | 5.20E-52 | CUL4B | cg12549513 | 0.68±0.07 | 0.77±0.11 | 5.45E-08 |
| ZNF6 | cg13082192 | 0.24±0.07 | 0.06±0.05 | 5.22E-52 | ZCCHC13 | cg03159836 | 0.64±0.09 | 0.76±0.15 | 6.93E-08 |
| IL1RAPL2 | cg22016571 | 0.25±0.07 | 0.08±0.04 | 7.45E-52 | TIMM17B | cg04323365 | 0.69±0.14 | 0.54±0.18 | 9.58E-08 |
| TMEM35 | cg15741706 | 0.35±0.08 | 0.16±0.05 | 9.20E-52 | OFD1 | cg23509027 | 0.58±0.15 | 0.43±0.17 | 1.24E-07 |
| FAM3A | cg22917082 | 0.36±0.1 | 0.09±0.07 | 9.25E-52 | TIMP1 | cg15812071 | 0.13±0.05 | 0.09±0.05 | 1.76E-07 |
| FLNA | cg02818322 | 0.41±0.13 | 0.1±0.08 | 1.58E-51 | GAGE2 | cg03704653 | 0.8±0.05 | 0.84±0.04 | 1.85E-07 |
| AR | cg26534002 | 0.34±0.1 | 0.08±0.07 | 2.24E-51 | FAM51A1 | cg08759036 | 0.12±0.11 | 0.07±0.04 | 1.88E-07 |
| BGN | cg18799866 | 0.56±0.09 | 0.27±0.08 | 2.28E-51 | HCFC1 | cg14321399 | 0.76±0.1 | 0.66±0.13 | 1.94E-07 |
| SUHW3 | cg06350796 | 0.26±0.08 | 0.06±0.05 | 2.45E-51 | OFD1 | cg04922020 | 0.56±0.14 | 0.44±0.15 | 2.17E-07 |
| RAB9B | cg19481052 | 0.33±0.1 | 0.07±0.07 | 6.07E-51 | HPRT1 | cg09920632 | 0.67±0.08 | 0.59±0.11 | 2.17E-07 |
| ZBTB33 | cg12537796 | 0.4±0.09 | 0.14±0.07 | 7.36E-51 | KAL1 | cg04211807 | 0.13±0.04 | 0.18±0.05 | 2.54E-07 |
| STK23 | cg11678767 | 0.58±0.13 | 0.22±0.1 | 1.07E-50 | S100G | cg00121904 | 0.59±0.09 | 0.52±0.09 | 2.81E-07 |
| EFNB1 | cg03595428 | 0.34±0.09 | 0.11±0.06 | 1.16E-50 | PLXNB3 | cg15465321 | 0.74±0.14 | 0.57±0.22 | 2.94E-07 |
| CXorf17 | cg12572939 | 0.32±0.08 | 0.11±0.06 | 1.87E-50 | OCRL | cg08023692 | 0.32±0.07 | 0.23±0.1 | 3.50E-07 |
| HADH2 | cg26833602 | 0.28±0.09 | 0.07±0.05 | 2.11E-50 | DRP2 | cg23026995 | 0.53±0.1 | 0.39±0.18 | 3.71E-07 |
| USP11 | cg24511534 | 0.42±0.11 | 0.12±0.08 | 2.30E-50 | ARSD | cg21567017 | 0.06±0.04 | 0.11±0.07 | 4.43E-07 |
| ITM2A | cg10246296 | 0.29±0.09 | 0.08±0.06 | 2.68E-50 | PNMA6A | cg01662650 | 0.9±0.04 | 0.85±0.07 | 5.49E-07 |
| FSHPRH1 | cg14179628 | 0.43±0.11 | 0.15±0.08 | 4.95E-50 | TIMP1 | cg13658777 | 0.24±0.06 | 0.17±0.08 | 5.65E-07 |
| C6orf68 | cg10078415 | 0.18±0.06 | 0.04±0.03 | 1.26E-49 | OPHN1 | cg16948369 | 0.09±0.06 | 0.05±0.05 | 6.61E-07 |
| RPS6KA3 | cg18923230 | 0.34±0.1 | 0.08±0.07 | 1.71E-49 | CXorf21 | cg01227519 | 0.48±0.09 | 0.38±0.15 | 6.68E-07 |
| LOC203427 | cg16429439 | 0.31±0.09 | 0.08±0.06 | 2.37E-49 | NLGN4X | cg23282949 | 0.12±0.05 | 0.16±0.06 | 7.15E-07 |
| RPL36A | cg26672426 | 0.37±0.12 | 0.08±0.08 | 2.51E-49 | LMO6 | cg09562455 | 0.79±0.06 | 0.67±0.16 | 7.87E-07 |
| BCAP31 | cg26617508 | 0.55±0.15 | 0.22±0.08 | 2.82E-49 | CXorf9 | cg03833774 | 0.66±0.13 | 0.49±0.24 | 8.20E-07 |
| MCTS1 | cg09676788 | 0.41±0.1 | 0.15±0.07 | 3.09E-49 | NUPL1 | cg06534422 | 0.22±0.06 | 0.17±0.05 | 8.88E-07 |
| RP2 | cg08254263 | 0.34±0.1 | 0.1±0.06 | 3.13E-49 | SSX6 | cg26798786 | 0.53±0.06 | 0.6±0.11 | 1.06E-06 |
| RBMX | cg14106263 | 0.44±0.12 | 0.14±0.08 | 3.38E-49 | ACE2 | cg07876586 | 0.66±0.1 | 0.57±0.13 | 1.35E-06 |
| MBTPS2 | cg26624294 | 0.41±0.1 | 0.16±0.07 | 3.46E-49 | TEX13B | cg24376537 | 0.77±0.05 | 0.82±0.06 | 1.36E-06 |
| GABRA3 | cg24183173 | 0.38±0.08 | 0.17±0.06 | 3.82E-49 | CLDN2 | cg16191875 | 0.66±0.11 | 0.54±0.16 | 1.48E-06 |
| TMEM29 | cg03037266 | 0.55±0.1 | 0.25±0.09 | 6.46E-49 | BCAP31 | cg24139739 | 0.79±0.04 | 0.75±0.05 | 1.62E-06 |
| FTSJ1 | cg04906538 | 0.26±0.08 | 0.09±0.04 | 1.17E-48 | NLGN3 | cg01776396 | 0.52±0.14 | 0.39±0.17 | 1.64E-06 |
| SLC9A6 | cg06208111 | 0.35±0.11 | 0.1±0.06 | 1.40E-48 | MAGEA11 | cg05508067 | 0.38±0.07 | 0.46±0.11 | 1.79E-06 |
| ABCD1 | cg17439480 | 0.31±0.08 | 0.1±0.06 | 1.60E-48 | ARMCX4 | cg13897449 | 0.54±0.07 | 0.45±0.13 | 1.88E-06 |
| PIGA | cg00941549 | 0.23±0.07 | 0.06±0.05 | 3.20E-48 | RP11-450P7.3 | cg03735049 | 0.6±0.09 | 0.49±0.15 | 1.97E-06 |
| PQBP1 | cg13918808 | 0.35±0.12 | 0.08±0.07 | 5.70E-48 | GDI1 | cg24103438 | 0.67±0.11 | 0.56±0.14 | 2.05E-06 |
| NSBP1 | cg01353347 | 0.26±0.07 | 0.08±0.05 | 6.66E-48 | WAS | cg26766480 | 0.55±0.1 | 0.43±0.18 | 2.16E-06 |
| IDS | cg13741802 | 0.29±0.1 | 0.07±0.06 | 7.34E-48 | NSDHL | cg09517019 | 0.61±0.15 | 0.48±0.17 | 2.27E-06 |
| MTCP1 | cg02804166 | 0.41±0.12 | 0.13±0.07 | 7.85E-48 | ALX4 | cg06654134 | 0.28±0.05 | 0.24±0.06 | 2.41E-06 |

- Sites highlighted in yellow locate in autosomes;
- Sites highlighted in red show sex differences in both peripheral blood cell and saliva DNA.
