# Supplementary material for: A Study of the Influence of Sex on Genome Wide Methylation
Source: PLoS One. 2010 Apr 6;5(4):e10028. doi: 10.1371/journal.pone.0010028 (PMC2850313; doi:10.1371/journal.pone.0010028)
Supplement: Table S2 — 580 autosomal sites showing sex difference on saliva DNA methylation. Some also show sex difference on peripheral blood cell DNA methylation. (0.66 MB DOC) [file pone.0010028.s002.doc]

Supporting Table S2: 580 autosomal sites showing sex difference on saliva DNA methylation

| Gene | Target ID | Chr. | Female  β-value | Male  β-value | P value |
| --- | --- | --- | --- | --- | --- |
| KCNA3 | cg00995520 | 1 | 0.28±0.08 | 0.25±0.07 | 3.55E-03 |
| KIAA0859 | cg01639034 | 1 | 0.09±0.03 | 0.11±0.06 | 1.89E-02 |
| INSRR | cg02049180 | 1 | 0.74±0.06 | 0.72±0.07 | 4.95E-02 |
| BCAN | cg02609880 | 1 | 0.33±0.05 | 0.31±0.04 | 4.26E-03 |
| ETNK2 | cg03718539 | 1 | 0.12±0.07 | 0.16±0.12 | 2.28E-02 |
| STMN1 | cg04471507 | 1 | 0.3±0.1 | 0.26±0.07 | 6.86E-04 |
| FLJ32784 | cg04637372 | 1 | 0.54±0.11 | 0.5±0.12 | 3.92E-02 |
| MRPS21 | cg05191071 | 1 | 0.11±0.04 | 0.13±0.06 | 2.61E-02 |
| C1orf65 | cg05333568 | 1 | 0.33±0.1 | 0.36±0.09 | 2.88E-02 |
| MOV10 | cg05587474 | 1 | 0.05±0.03 | 0.07±0.05 | 3.80E-02 |
| C4BPB | cg05659947 | 1 | 0.72±0.07 | 0.69±0.09 | 3.55E-02 |
| CR1 | cg05825950 | 1 | 0.11±0.04 | 0.13±0.06 | 3.51E-02 |
| GRIK3 | cg06165395 | 1 | 0.13±0.03 | 0.14±0.03 | 4.21E-02 |
| LRRC8C | cg06641366 | 1 | 0.84±0.06 | 0.82±0.07 | 2.43E-02 |
| BMP8B | cg07082331 | 1 | 0.07±0.02 | 0.08±0.04 | 1.47E-02 |
| NDUFS5 | cg07388493 | 1 | 0.54±0.06 | 0.51±0.07 | 2.51E-02 |
| GJA5 | cg08307963 | 1 | 0.1±0.05 | 0.12±0.06 | 1.04E-02 |
| APITD1 | cg09242541 | 1 | 0.42±0.05 | 0.4±0.06 | 8.84E-03 |
| TDRD5 | cg09656934 | 1 | 0.34±0.09 | 0.3±0.08 | 1.47E-03 |
| OPRD1 | cg09970593 | 1 | 0.71±0.08 | 0.68±0.09 | 4.01E-02 |
| LAMC1 | cg10847798 | 1 | 0.08±0.03 | 0.09±0.05 | 2.11E-02 |
| KIAA2013 | cg11345438 | 1 | 0.65±0.08 | 0.68±0.08 | 1.67E-02 |
| ARID1A | cg11856093 | 1 | 0.1±0.05 | 0.12±0.08 | 2.48E-02 |
| BGLAP | cg13282594 | 1 | 0.64±0.05 | 0.67±0.05 | 3.45E-03 |
| HSPA6 | cg13817266 | 1 | 0.35±0.09 | 0.32±0.1 | 4.30E-02 |
| ANP32E | cg13841804 | 1 | 0.12±0.04 | 0.14±0.06 | 1.49E-02 |
| RPS6KC1 | cg14576824 | 1 | 0.38±0.07 | 0.33±0.08 | 4.61E-04 |
| AMY2A | cg15573237 | 1 | 0.61±0.04 | 0.59±0.04 | 4.61E-02 |
| TSSK3 | cg16379513 | 1 | 0.83±0.07 | 0.8±0.09 | 2.08E-02 |
| CACYBP | cg16743289 | 1 | 0.49±0.08 | 0.45±0.09 | 1.30E-02 |
| ACTN2 | cg16853982 | 1 | 0.25±0.07 | 0.23±0.06 | 3.18E-02 |
| BARHL2 | cg17241310 | 1 | 0.16±0.07 | 0.19±0.1 | 4.76E-02 |
| SYCP1 | cg18087477 | 1 | 0.44±0.06 | 0.4±0.06 | 1.01E-04 |
| SERBP1 | cg18259391 | 1 | 0.24±0.04 | 0.21±0.04 | 1.34E-04 |
| OBSCN | cg19109050 | 1 | 0.71±0.08 | 0.68±0.08 | 1.40E-02 |
| FLJ46380 | cg19180828 | 1 | 0.34±0.08 | 0.31±0.07 | 2.17E-03 |
| RYR2 | cg19764418 | 1 | 0.53±0.09 | 0.47±0.12 | 3.07E-03 |
| B3GALT6 | cg19945840 | 1 | 0.43±0.06 | 0.46±0.05 | 2.34E-03 |
| NPPA | cg20202438 | 1 | 0.8±0.04 | 0.82±0.03 | 1.21E-02 |
| PEX10 | cg20664247 | 1 | 0.6±0.12 | 0.55±0.15 | 2.99E-02 |
| ACTN2 | cg21376883 | 1 | 0.19±0.05 | 0.17±0.04 | 1.79E-03 |
| ACOT11 | cg21448423 | 1 | 0.38±0.07 | 0.4±0.06 | 2.53E-02 |
| DNALI1 | cg21488617 | 1 | 0.41±0.06 | 0.38±0.07 | 3.13E-02 |
| DIRAS3 | cg21808053 | 1 | 0.5±0.06 | 0.48±0.07 | 3.93E-02 |
| MSH4 | cg22035229 | 1 | 0.84±0.1 | 0.8±0.12 | 3.54E-02 |
| STMN1 | cg23323671 | 1 | 0.39±0.1 | 0.36±0.09 | 1.46E-02 |
| FAM19A3 | cg23746359 | 1 | 0.45±0.08 | 0.43±0.08 | 4.54E-02 |
| PPIE | cg24920358 | 1 | 0.39±0.09 | 0.36±0.09 | 4.34E-02 |
| S100A4 | cg26894575 | 1 | 0.04±0.02 | 0.06±0.04 | 2.66E-02 |
| HAPLN2 | cg27138018 | 1 | 0.13±0.04 | 0.14±0.05 | 4.45E-02 |
| YBX1 | cg27530424 | 1 | 0.46±0.05 | 0.49±0.06 | 2.11E-03 |
| STK40 | cg27546682 | 1 | 0.06±0.03 | 0.07±0.04 | 1.22E-02 |
| FLJ25084 | cg02803836 | 2 | 0.87±0.08 | 0.83±0.13 | 4.08E-02 |
| FLJ20701 | cg04180868 | 2 | 0.52±0.07 | 0.54±0.08 | 4.73E-02 |
| JMJD1A | cg04590978 | 2 | 0.07±0.02 | 0.08±0.04 | 4.17E-02 |
| NR4A2 | cg06139836 | 2 | 0.04±0.03 | 0.06±0.05 | 3.33E-02 |
| GALM | cg08012454 | 2 | 0.12±0.05 | 0.14±0.06 | 4.90E-02 |
| APOB | cg08158570 | 2 | 0.18±0.07 | 0.15±0.06 | 2.02E-02 |
| CGREF1 | cg09260089 | 2 | 0.48±0.04 | 0.46±0.05 | 1.25E-03 |
| ZNF512 | cg10234776 | 2 | 0.14±0.06 | 0.17±0.07 | 3.54E-02 |
| NDUFS1 | cg10247268 | 2 | 0.31±0.05 | 0.28±0.06 | 1.29E-04 |
| STEAP3 | cg10367730 | 2 | 0.09±0.04 | 0.11±0.05 | 2.05E-02 |
| INHBB | cg10983208 | 2 | 0.08±0.03 | 0.1±0.05 | 4.35E-02 |
| PAX8 | cg11438755 | 2 | 0.75±0.04 | 0.74±0.04 | 4.37E-02 |
| ZNF512 | cg12087643 | 2 | 0.09±0.03 | 0.1±0.04 | 4.91E-02 |
| POMC | cg12230010 | 2 | 0.33±0.11 | 0.3±0.1 | 2.78E-02 |
| ANKMY1 | cg12621514 | 2 | 0.1±0.08 | 0.14±0.09 | 1.15E-02 |
| SUPT7L | cg13548361 | 2 | 0.07±0.03 | 0.09±0.04 | 3.80E-02 |
| LIPT1 | cg14540150 | 2 | 0.07±0.03 | 0.08±0.04 | 4.63E-02 |
| FAM49A | cg14826456 | 2 | 0.34±0.09 | 0.31±0.1 | 4.84E-02 |
| ALS2CR11 | cg16933922 | 2 | 0.35±0.09 | 0.31±0.09 | 1.35E-02 |
| DKFZp434O0527 | cg21033494 | 2 | 0.81±0.05 | 0.82±0.03 | 4.99E-02 |
| SCTR | cg24631950 | 2 | 0.13±0.03 | 0.12±0.03 | 1.01E-02 |
| ARHGAP25 | cg24668150 | 2 | 0.19±0.06 | 0.24±0.1 | 2.76E-03 |
| ALS2CR11 | cg25833031 | 2 | 0.52±0.1 | 0.47±0.11 | 1.18E-02 |
| TACSTD1 | cg26477793 | 2 | 0.4±0.13 | 0.34±0.13 | 1.09E-02 |
| FLJ13391 | cg26644395 | 2 | 0.77±0.07 | 0.74±0.1 | 1.26E-02 |
| UBE2F | cg26778908 | 2 | 0.08±0.03 | 0.09±0.05 | 4.86E-02 |
| CFC1 | cg01987509 | 2 | 0.32±0.09 | 0.36±0.11 | 4.78E-02 |
| ACYP2 | cg02005336 | 2 | 0.3±0.11 | 0.36±0.13 | 7.70E-03 |
| GTF3C3 | cg02254407 | 2 | 0.87±0.04 | 0.85±0.04 | 1.36E-03 |
| SLC9A2 | cg03160508 | 2 | 0.39±0.13 | 0.3±0.12 | 2.50E-05 |
| R3HDM1 | cg03222066 | 2 | 0.08±0.04 | 0.1±0.07 | 2.37E-02 |
| RPL31 | cg05676042 | 2 | 0.53±0.09 | 0.5±0.09 | 2.18E-02 |
| PDE1A | cg09541468 | 2 | 0.59±0.07 | 0.62±0.09 | 2.06E-02 |
| LRPPRC | cg09555217 | 2 | 0.07±0.05 | 0.1±0.08 | 7.78E-03 |
| CHRNG | cg10467098 | 2 | 0.27±0.09 | 0.25±0.09 | 3.52E-02 |
| ACPL2 | cg10977115 | 3 | 0.24±0.08 | 0.28±0.11 | 3.81E-02 |
| TMEM22 | cg12907644 | 3 | 0.26±0.11 | 0.31±0.14 | 2.18E-02 |
| C3orf31 | cg13250711 | 3 | 0.06±0.04 | 0.08±0.08 | 3.84E-02 |
| CD96 | cg13864937 | 3 | 0.33±0.06 | 0.31±0.07 | 4.33E-02 |
| MYLK | cg14430111 | 3 | 0.36±0.05 | 0.33±0.06 | 1.91E-02 |
| ZNF501 | cg14464791 | 3 | 0.2±0.04 | 0.18±0.05 | 1.76E-02 |
| PODXL2 | cg14775560 | 3 | 0.23±0.09 | 0.19±0.07 | 2.79E-03 |
| MINA | cg14967066 | 3 | 0.16±0.04 | 0.19±0.06 | 1.79E-03 |
| SPATA16 | cg16404106 | 3 | 0.73±0.05 | 0.71±0.07 | 1.02E-02 |
| RASSF1 | cg16588061 | 3 | 0.16±0.06 | 0.22±0.1 | 1.59E-04 |
| RASSF1 | cg16612699 | 3 | 0.3±0.1 | 0.37±0.14 | 6.55E-04 |
| WDR52 | cg16668394 | 3 | 0.29±0.06 | 0.26±0.07 | 1.28E-02 |
| LRRC2 | cg17074212 | 3 | 0.75±0.05 | 0.82±0.04 | 3.27E-23 |
| HRASLS | cg17838516 | 3 | 0.13±0.06 | 0.16±0.08 | 1.31E-02 |
| MCCC1 | cg17838765 | 3 | 0.21±0.14 | 0.28±0.19 | 2.85E-02 |
| ILDR1 | cg17891278 | 3 | 0.29±0.08 | 0.26±0.09 | 3.77E-02 |
| CPNE4 | cg18538812 | 3 | 0.13±0.07 | 0.16±0.08 | 1.76E-02 |
| NCK1 | cg19017420 | 3 | 0.12±0.05 | 0.14±0.07 | 3.77E-02 |
| TDGF1 | cg19327844 | 3 | 0.51±0.07 | 0.64±0.06 | 2.85E-28 |
| PLOD2 | cg19596755 | 3 | 0.24±0.06 | 0.19±0.06 | 8.57E-06 |
| KNG1 | cg21096399 | 3 | 0.45±0.09 | 0.41±0.1 | 9.60E-03 |
| GP5 | cg21759080 | 3 | 0.51±0.1 | 0.54±0.09 | 1.65E-02 |
| CPA3 | cg21939221 | 3 | 0.45±0.08 | 0.42±0.07 | 4.08E-02 |
| ATP11B | cg22424444 | 3 | 0.06±0.03 | 0.07±0.04 | 3.27E-02 |
| MITF | cg22635155 | 3 | 0.06±0.02 | 0.07±0.04 | 1.48E-02 |
| SOX2 | cg23517677 | 3 | 0.21±0.06 | 0.19±0.05 | 3.69E-02 |
| CSPG5 | cg24625128 | 3 | 0.37±0.07 | 0.34±0.07 | 4.52E-03 |
| TSP50 | cg27497687 | 3 | 0.48±0.11 | 0.55±0.11 | 4.61E-05 |
| TOMM70A | cg01097940 | 3 | 0.15±0.12 | 0.2±0.13 | 2.94E-02 |
| PH-4 | cg01255349 | 3 | 0.19±0.08 | 0.22±0.1 | 2.60E-02 |
| LMLN | cg01376672 | 3 | 0.77±0.08 | 0.74±0.1 | 3.54E-02 |
| NR2C2 | cg01657380 | 3 | 0.17±0.06 | 0.19±0.08 | 4.72E-02 |
| CHST13 | cg03310453 | 3 | 0.32±0.06 | 0.29±0.07 | 2.01E-02 |
| RPP14 | cg05722918 | 3 | 0.11±0.03 | 0.12±0.05 | 3.73E-02 |
| DRD3 | cg06417225 | 3 | 0.24±0.04 | 0.25±0.04 | 2.56E-02 |
| TIMP4 | cg06462703 | 3 | 0.24±0.08 | 0.2±0.08 | 8.83E-03 |
| MCF2L2 | cg07618900 | 3 | 0.09±0.04 | 0.11±0.06 | 2.33E-02 |
| CYTL1 | cg08900043 | 4 | 0.12±0.05 | 0.17±0.07 | 1.82E-05 |
| GAB1 | cg09163317 | 4 | 0.41±0.07 | 0.38±0.08 | 2.17E-02 |
| MSX1 | cg09239756 | 4 | 0.22±0.06 | 0.25±0.08 | 1.64E-02 |
| FAT | cg10141715 | 4 | 0.1±0.05 | 0.12±0.06 | 4.13E-02 |
| FLJ11017 | cg11065385 | 4 | 0.17±0.07 | 0.2±0.08 | 3.11E-02 |
| TMSL3 | cg12391174 | 4 | 0.6±0.06 | 0.64±0.07 | 8.00E-04 |
| OTOP1 | cg13236637 | 4 | 0.75±0.05 | 0.77±0.05 | 1.32E-02 |
| FLJ20032 | cg14059963 | 4 | 0.11±0.03 | 0.13±0.04 | 2.74E-02 |
| UGDH | cg14217534 | 4 | 0.16±0.08 | 0.12±0.07 | 1.84E-04 |
| KLF3 | cg14817848 | 4 | 0.21±0.07 | 0.24±0.09 | 2.91E-02 |
| GRSF1 | cg16807911 | 4 | 0.08±0.03 | 0.09±0.04 | 3.12E-02 |
| SLC25A31 | cg17416608 | 4 | 0.94±0.04 | 0.92±0.05 | 1.78E-02 |
| EPHA5 | cg17469479 | 4 | 0.06±0.03 | 0.08±0.05 | 1.20E-02 |
| FLJ35725 | cg18081313 | 4 | 0.83±0.12 | 0.78±0.16 | 4.97E-02 |
| HHIP | cg18124330 | 4 | 0.05±0.02 | 0.06±0.04 | 2.20E-02 |
| OTOP1 | cg18344063 | 4 | 0.2±0.05 | 0.24±0.07 | 7.33E-06 |
| PF4 | cg18470891 | 4 | 0.55±0.14 | 0.5±0.13 | 2.55E-02 |
| EVC2 | cg18628483 | 4 | 0.19±0.05 | 0.17±0.05 | 7.36E-03 |
| PCGF3 | cg19020694 | 4 | 0.26±0.06 | 0.24±0.06 | 2.53E-02 |
| SULT1B1 | cg19434066 | 4 | 0.52±0.04 | 0.5±0.06 | 2.71E-02 |
| MGC46496 | cg20088913 | 4 | 0.15±0.05 | 0.14±0.04 | 4.63E-02 |
| LOC92345 | cg20424530 | 4 | 0.81±0.04 | 0.79±0.05 | 9.19E-03 |
| EPHA5 | cg20673611 | 4 | 0.07±0.04 | 0.09±0.05 | 3.70E-02 |
| FGF5 | cg20784775 | 4 | 0.1±0.04 | 0.12±0.04 | 3.43E-02 |
| MGC27016 | cg23495733 | 4 | 0.74±0.1 | 0.71±0.09 | 3.35E-02 |
| FLJ23191 | cg25181284 | 4 | 0.13±0.06 | 0.15±0.07 | 4.66E-02 |
| GDEP | cg25605408 | 4 | 0.84±0.04 | 0.81±0.07 | 5.70E-03 |
| ACOT12 | cg26956535 | 5 | 0.26±0.09 | 0.23±0.05 | 2.15E-03 |
| PCDHGB4 | cg27188703 | 5 | 0.12±0.03 | 0.14±0.03 | 1.34E-03 |
| APC | cg27394563 | 5 | 0.36±0.05 | 0.39±0.05 | 4.83E-05 |
| FST | cg27476329 | 5 | 0.08±0.04 | 0.09±0.05 | 4.08E-02 |
| PCDHB3 | cg00109274 | 5 | 0.35±0.07 | 0.37±0.06 | 4.93E-02 |
| PCDHB8 | cg01679262 | 5 | 0.17±0.04 | 0.19±0.05 | 2.57E-02 |
| EBF | cg09185773 | 5 | 0.12±0.03 | 0.13±0.03 | 7.60E-03 |
| PAPD4 | cg09646392 | 5 | 0.1±0.03 | 0.11±0.05 | 2.37E-02 |
| LHFPL2 | cg12582008 | 5 | 0.26±0.07 | 0.29±0.08 | 2.73E-02 |
| CSF1R | cg13614181 | 5 | 0.8±0.11 | 0.76±0.14 | 3.90E-02 |
| HIGD2A | cg14405589 | 5 | 0.19±0.05 | 0.21±0.06 | 2.75E-02 |
| RAB9P1 | cg23934633 | 5 | 0.76±0.05 | 0.63±0.06 | 8.68E-33 |
| OCLN | cg01419788 | 5 | 0.1±0.03 | 0.12±0.06 | 3.96E-02 |
| PCDHB13 | cg02629761 | 5 | 0.1±0.03 | 0.11±0.04 | 3.21E-02 |
| KENAE | cg03366677 | 5 | 0.13±0.07 | 0.11±0.05 | 3.69E-02 |
| PCDHGC4 | cg04269351 | 5 | 0.07±0.02 | 0.08±0.03 | 3.75E-02 |
| CDX1 | cg04394967 | 5 | 0.55±0.09 | 0.52±0.09 | 2.61E-02 |
| LTC4S | cg05923056 | 5 | 0.12±0.04 | 0.14±0.05 | 2.41E-02 |
| NEUROG1 | cg08020808 | 5 | 0.12±0.03 | 0.13±0.04 | 1.94E-02 |
| PCDHA9 | cg11490446 | 5 | 0.54±0.07 | 0.51±0.08 | 1.75E-02 |
| CENPH | cg13438944 | 5 | 0.43±0.07 | 0.41±0.07 | 4.93E-02 |
| UGT3A1 | cg15078479 | 5 | 0.14±0.03 | 0.15±0.04 | 2.01E-02 |
| BASP1 | cg15186869 | 5 | 0.1±0.04 | 0.12±0.07 | 3.54E-02 |
| TRIP13 | cg15301694 | 5 | 0.56±0.09 | 0.6±0.11 | 3.15E-02 |
| LMNB1 | cg15492104 | 5 | 0.13±0.08 | 0.17±0.13 | 1.27E-02 |
| P4HA2 | cg15677132 | 5 | 0.45±0.13 | 0.4±0.14 | 2.52E-02 |
| FGD2 | cg20337385 | 6 | 0.88±0.07 | 0.83±0.1 | 2.73E-03 |
| RNF8 | cg23709121 | 6 | 0.66±0.07 | 0.62±0.08 | 5.97E-04 |
| DAAM2 | cg24058132 | 6 | 0.47±0.05 | 0.45±0.06 | 2.83E-02 |
| SLC29A1 | cg24121436 | 6 | 0.44±0.1 | 0.48±0.11 | 3.94E-02 |
| PCMT1 | cg24165760 | 6 | 0.04±0.02 | 0.05±0.04 | 1.80E-02 |
| PRSS16 | cg25906332 | 6 | 0.57±0.12 | 0.52±0.12 | 1.34E-02 |
| HIST1H4A | cg27504299 | 6 | 0.13±0.07 | 0.16±0.1 | 4.55E-02 |
| CRISP2 | cg00964109 | 6 | 0.68±0.11 | 0.6±0.12 | 7.15E-06 |
| BXDC1 | cg01989224 | 6 | 0.45±0.15 | 0.39±0.16 | 6.77E-03 |
| NOTCH4 | cg03377682 | 6 | 0.52±0.09 | 0.47±0.08 | 2.69E-04 |
| LSM2 | cg06958211 | 6 | 0.12±0.04 | 0.14±0.06 | 4.68E-02 |
| DDX43 | cg07501965 | 6 | 0.75±0.07 | 0.69±0.08 | 3.28E-05 |
| HIST1H2AG | cg08291539 | 6 | 0.11±0.06 | 0.14±0.09 | 2.45E-02 |
| CRISP2 | cg08780378 | 6 | 0.64±0.14 | 0.56±0.18 | 2.06E-03 |
| COL11A2 | cg11015241 | 6 | 0.19±0.05 | 0.22±0.08 | 1.76E-02 |
| HIST1H4L | cg11611600 | 6 | 0.2±0.08 | 0.24±0.1 | 1.60E-02 |
| C6orf106 | cg15089487 | 6 | 0.14±0.06 | 0.16±0.08 | 4.91E-02 |
| PLAGL1 | cg15198335 | 6 | 0.74±0.07 | 0.76±0.08 | 4.54E-02 |
| SPACA1 | cg16711185 | 6 | 0.75±0.09 | 0.7±0.09 | 2.15E-03 |
| DTNBP1 | cg19911362 | 6 | 0.68±0.06 | 0.65±0.06 | 8.74E-04 |
| DDX43 | cg23318804 | 6 | 0.89±0.06 | 0.86±0.08 | 1.65E-03 |
| PPT2 | cg23393291 | 6 | 0.26±0.06 | 0.23±0.06 | 6.93E-03 |
| DDAH2 | cg24447890 | 6 | 0.2±0.09 | 0.24±0.13 | 3.76E-02 |
| MICAL1 | cg04413148 | 6 | 0.09±0.04 | 0.11±0.06 | 2.59E-02 |
| TCF21 | cg05732130 | 6 | 0.32±0.05 | 0.35±0.07 | 1.27E-02 |
| CDSN | cg06320982 | 6 | 0.35±0.07 | 0.38±0.08 | 4.06E-02 |
| C6orf68 | cg10078415 | 6 | 0.18±0.06 | 0.04±0.03 | 1.26E-49 |
| SEC61G | cg11869721 | 7 | 0.21±0.07 | 0.17±0.07 | 1.41E-03 |
| UBE3C | cg12879297 | 7 | 0.18±0.07 | 0.16±0.06 | 4.72E-02 |
| TRIM50C | cg14696348 | 7 | 0.59±0.15 | 0.51±0.18 | 5.52E-03 |
| HOXA6 | cg14959707 | 7 | 0.06±0.02 | 0.07±0.02 | 7.89E-03 |
| DBF4 | cg15309006 | 7 | 0.14±0.04 | 0.15±0.05 | 4.81E-02 |
| FIGNL1 | cg15834294 | 7 | 0.44±0.07 | 0.4±0.07 | 1.70E-04 |
| IMPDH1 | cg15890111 | 7 | 0.06±0.03 | 0.08±0.06 | 7.82E-03 |
| PEG10 | cg17698643 | 7 | 0.62±0.04 | 0.64±0.04 | 1.95E-03 |
| MKRN1 | cg17745122 | 7 | 0.15±0.04 | 0.12±0.04 | 2.83E-05 |
| HSPC268 | cg18711066 | 7 | 0.05±0.02 | 0.06±0.04 | 2.17E-02 |
| PARP12 | cg19091641 | 7 | 0.49±0.05 | 0.45±0.05 | 1.76E-05 |
| LOC168850 | cg19297232 | 7 | 0.26±0.05 | 0.24±0.04 | 2.50E-02 |
| EPO | cg19703826 | 7 | 0.09±0.03 | 0.11±0.04 | 4.06E-02 |
| FLJ12700 | cg20047230 | 7 | 0.41±0.05 | 0.39±0.05 | 1.81E-02 |
| PAX4 | cg20253700 | 7 | 0.76±0.05 | 0.74±0.06 | 2.41E-02 |
| HOXA2 | cg21184011 | 7 | 0.2±0.04 | 0.22±0.05 | 8.31E-03 |
| CDK5 | cg22677246 | 7 | 0.09±0.04 | 0.11±0.07 | 1.21E-02 |
| ACTL6B | cg25898500 | 7 | 0.5±0.14 | 0.45±0.14 | 4.70E-02 |
| MET | cg26134665 | 7 | 0.09±0.03 | 0.1±0.04 | 4.71E-02 |
| IMPDH1 | cg00031162 | 7 | 0.05±0.03 | 0.07±0.06 | 3.17E-02 |
| SP8 | cg02949544 | 7 | 0.23±0.04 | 0.24±0.04 | 3.90E-02 |
| POR | cg03192551 | 7 | 0.33±0.13 | 0.37±0.16 | 4.99E-02 |
| FLJ39237 | cg04518106 | 7 | 0.44±0.09 | 0.4±0.08 | 1.53E-03 |
| PDK4 | cg04532952 | 7 | 0.3±0.06 | 0.27±0.07 | 3.00E-02 |
| PON3 | cg06374075 | 7 | 0.16±0.06 | 0.14±0.06 | 1.56E-02 |
| HOXA9 | cg06537829 | 7 | 0.17±0.06 | 0.19±0.07 | 2.81E-02 |
| HOXA2 | cg07126839 | 7 | 0.21±0.04 | 0.24±0.05 | 3.15E-03 |
| HCA112 | cg07292816 | 7 | 0.28±0.07 | 0.24±0.08 | 1.16E-03 |
| HOXA7 | cg07471052 | 7 | 0.2±0.07 | 0.24±0.09 | 4.88E-03 |
| NPBWR1 | cg09978128 | 8 | 0.06±0.02 | 0.07±0.03 | 3.07E-02 |
| C8orf31 | cg11221508 | 8 | 0.53±0.12 | 0.49±0.11 | 1.93E-02 |
| FOXH1 | cg11816577 | 8 | 0.79±0.09 | 0.76±0.1 | 3.82E-02 |
| WRN | cg14314889 | 8 | 0.13±0.03 | 0.15±0.05 | 1.61E-02 |
| PLAT | cg15206330 | 8 | 0.33±0.09 | 0.3±0.09 | 4.11E-02 |
| SCARA3 | cg15994159 | 8 | 0.27±0.06 | 0.26±0.05 | 3.52E-02 |
| GATA4 | cg16585619 | 8 | 0.11±0.03 | 0.12±0.03 | 2.21E-02 |
| DENND3 | cg18062196 | 8 | 0.08±0.04 | 0.09±0.06 | 3.38E-02 |
| TRAM1 | cg18536148 | 8 | 0.25±0.07 | 0.21±0.06 | 1.19E-03 |
| PIWIL2 | cg19267846 | 8 | 0.83±0.09 | 0.79±0.1 | 1.20E-02 |
| TPD52 | cg19566405 | 8 | 0.19±0.06 | 0.22±0.07 | 1.62E-02 |
| CRH | cg19668255 | 8 | 0.61±0.11 | 0.57±0.13 | 4.43E-02 |
| CTHRC1 | cg20104688 | 8 | 0.87±0.04 | 0.85±0.04 | 2.80E-02 |
| LACTB2 | cg20406482 | 8 | 0.42±0.1 | 0.38±0.12 | 3.86E-02 |
| CA2 | cg20551517 | 8 | 0.13±0.06 | 0.17±0.1 | 2.64E-03 |
| GATA4 | cg20612559 | 8 | 0.13±0.03 | 0.14±0.03 | 1.63E-02 |
| PABPC1 | cg20657421 | 8 | 0.25±0.06 | 0.23±0.07 | 2.71E-02 |
| DPYS | cg20770803 | 8 | 0.4±0.06 | 0.37±0.06 | 4.29E-04 |
| GPR172A | cg21391660 | 8 | 0.29±0.04 | 0.3±0.05 | 4.43E-02 |
| STMN2 | cg22264436 | 8 | 0.2±0.04 | 0.22±0.04 | 1.40E-02 |
| ZNF572 | cg22968401 | 8 | 0.84±0.03 | 0.83±0.04 | 3.60E-02 |
| FGF20 | cg22983092 | 8 | 0.09±0.03 | 0.11±0.04 | 4.30E-02 |
| HHCM | cg23267110 | 8 | 0.26±0.06 | 0.24±0.07 | 1.67E-02 |
| EXTL3 | cg23906144 | 8 | 0.22±0.08 | 0.26±0.11 | 4.45E-02 |
| TRAM1 | cg24073161 | 8 | 0.48±0.1 | 0.44±0.1 | 3.94E-03 |
| TNFRSF10A | cg24357161 | 8 | 0.81±0.08 | 0.78±0.11 | 3.63E-02 |
| LY6K | cg24456340 | 8 | 0.44±0.14 | 0.39±0.13 | 1.10E-02 |
| FLJ20444 | cg25949363 | 9 | 0.25±0.09 | 0.31±0.09 | 2.29E-05 |
| C9orf64 | cg26771272 | 9 | 0.36±0.1 | 0.31±0.1 | 2.69E-03 |
| ZFP37 | cg27178345 | 9 | 0.24±0.08 | 0.19±0.06 | 2.04E-05 |
| GPR21 | cg01794711 | 9 | 0.23±0.08 | 0.27±0.1 | 1.66E-02 |
| MGC42630 | cg03468463 | 9 | 0.71±0.06 | 0.68±0.08 | 2.54E-02 |
| FANCG | cg03813215 | 9 | 0.69±0.1 | 0.63±0.11 | 1.56E-03 |
| SLC31A2 | cg04564030 | 9 | 0.28±0.06 | 0.26±0.06 | 1.64E-02 |
| PGM5 | cg05637351 | 9 | 0.24±0.08 | 0.21±0.08 | 1.21E-02 |
| C9orf84 | cg06804711 | 9 | 0.85±0.07 | 0.81±0.11 | 7.60E-03 |
| FXN | cg07851192 | 9 | 0.32±0.05 | 0.3±0.05 | 2.96E-02 |
| BAG1 | cg09130402 | 9 | 0.79±0.08 | 0.82±0.1 | 3.40E-02 |
| PRAF1 | cg19496782 | 9 | 0.11±0.04 | 0.09±0.03 | 4.43E-02 |
| C9orf112 | cg24362401 | 9 | 0.42±0.1 | 0.39±0.1 | 4.07E-02 |
| C9orf84 | cg26755793 | 9 | 0.66±0.09 | 0.62±0.1 | 2.15E-02 |
| SUSD1 | cg00643392 | 9 | 0.15±0.11 | 0.21±0.15 | 1.25E-02 |
| SURF6 | cg00698688 | 9 | 0.11±0.05 | 0.08±0.04 | 2.04E-05 |
| TLE1 | cg01454134 | 9 | 0.47±0.12 | 0.09±0.07 | 6.49E-69 |
| GRIN1 | cg03257423 | 9 | 0.25±0.04 | 0.27±0.04 | 1.27E-02 |
| CDKN2B | cg03289872 | 9 | 0.08±0.04 | 0.1±0.04 | 4.78E-02 |
| TRPM3 | cg03747695 | 9 | 0.09±0.04 | 0.11±0.06 | 3.85E-02 |
| REXO4 | cg04322978 | 9 | 0.58±0.05 | 0.6±0.06 | 2.99E-02 |
| ZDHHC12 | cg05023116 | 9 | 0.11±0.04 | 0.13±0.05 | 2.06E-02 |
| MGC41945 | cg05294243 | 9 | 0.49±0.08 | 0.46±0.09 | 1.51E-02 |
| DNAI1 | cg05346899 | 9 | 0.61±0.08 | 0.57±0.13 | 3.56E-02 |
| FREQ | cg05467458 | 9 | 0.2±0.06 | 0.17±0.07 | 7.21E-03 |
| HDHD3 | cg05508084 | 9 | 0.47±0.07 | 0.45±0.08 | 3.56E-02 |
| CYP2E1 | cg07608333 | 10 | 0.65±0.15 | 0.6±0.16 | 4.79E-02 |
| CNNM1 | cg08458170 | 10 | 0.2±0.06 | 0.19±0.04 | 2.40E-02 |
| PTPN20B | cg08607082 | 10 | 0.66±0.13 | 0.58±0.14 | 4.67E-04 |
| MGMT | cg08684473 | 10 | 0.7±0.09 | 0.66±0.11 | 4.73E-02 |
| LBX1 | cg09227563 | 10 | 0.07±0.03 | 0.08±0.04 | 4.39E-02 |
| C10orf93 | cg09251995 | 10 | 0.09±0.04 | 0.11±0.05 | 2.22E-02 |
| BMS1L | cg10480741 | 10 | 0.26±0.08 | 0.22±0.08 | 1.12E-03 |
| MGMT | cg11834681 | 10 | 0.75±0.11 | 0.71±0.12 | 4.88E-02 |
| HHEX | cg13282951 | 10 | 0.18±0.05 | 0.2±0.06 | 2.24E-02 |
| NRP1 | cg13438673 | 10 | 0.09±0.06 | 0.13±0.1 | 1.73E-03 |
| GLUD1 | cg14271499 | 10 | 0.44±0.08 | 0.27±0.06 | 4.61E-42 |
| MPP7 | cg15177917 | 10 | 0.75±0.07 | 0.72±0.11 | 3.68E-02 |
| TCF7L2 | cg15984661 | 10 | 0.08±0.03 | 0.09±0.05 | 4.57E-02 |
| KAZALD1 | cg16481281 | 10 | 0.79±0.07 | 0.82±0.06 | 4.34E-03 |
| HNRPF | cg16483916 | 10 | 0.24±0.05 | 0.22±0.06 | 2.87E-02 |
| GSTO2 | cg16490390 | 10 | 0.07±0.04 | 0.1±0.06 | 1.25E-02 |
| CNNM2 | cg16519742 | 10 | 0.26±0.06 | 0.28±0.07 | 2.57E-02 |
| PTPN20B | cg16749578 | 10 | 0.54±0.16 | 0.46±0.16 | 1.41E-03 |
| XPNPEP1 | cg17226343 | 10 | 0.41±0.09 | 0.38±0.1 | 3.65E-02 |
| C10orf82 | cg17389295 | 10 | 0.65±0.1 | 0.62±0.09 | 4.25E-02 |
| DYDC1 | cg17847607 | 10 | 0.28±0.08 | 0.25±0.09 | 2.36E-02 |
| WDR37 | cg18828334 | 10 | 0.2±0.08 | 0.24±0.11 | 4.77E-02 |
| DUSP5 | cg19147390 | 10 | 0.4±0.11 | 0.37±0.11 | 4.75E-02 |
| RPP38 | cg19995014 | 10 | 0.07±0.04 | 0.09±0.05 | 1.40E-02 |
| SORBS1 | cg21252483 | 10 | 0.26±0.06 | 0.24±0.05 | 2.86E-02 |
| SFRP5 | cg21331821 | 10 | 0.27±0.07 | 0.25±0.06 | 1.76E-02 |
| ZNF485 | cg21550442 | 10 | 0.56±0.06 | 0.55±0.05 | 4.96E-02 |
| MGMT | cg21663431 | 10 | 0.36±0.08 | 0.31±0.09 | 2.58E-04 |
| DLG2 | cg23404467 | 11 | 0.33±0.08 | 0.3±0.08 | 2.21E-02 |
| BTG4 | cg23824815 | 11 | 0.12±0.04 | 0.13±0.04 | 1.80E-02 |
| SSRP1 | cg24056562 | 11 | 0.15±0.04 | 0.13±0.04 | 3.48E-02 |
| WT1 | cg24311282 | 11 | 0.39±0.1 | 0.34±0.1 | 6.70E-03 |
| SLC22A18AS | cg25234963 | 11 | 0.72±0.12 | 0.66±0.14 | 1.15E-02 |
| GPR44 | cg25397562 | 11 | 0.17±0.07 | 0.19±0.07 | 4.22E-02 |
| ABCG4 | cg25622628 | 11 | 0.43±0.06 | 0.4±0.06 | 2.27E-03 |
| MAPK8IP1 | cg25999015 | 11 | 0.19±0.04 | 0.21±0.05 | 3.45E-02 |
| WT1 | cg26626089 | 11 | 0.31±0.09 | 0.27±0.1 | 2.02E-02 |
| IGF2AS | cg26646370 | 11 | 0.18±0.05 | 0.2±0.06 | 4.21E-02 |
| SLC22A18 | cg27285056 | 11 | 0.78±0.07 | 0.8±0.06 | 1.44E-02 |
| P2RY6 | cg01600782 | 11 | 0.28±0.1 | 0.25±0.09 | 1.83E-02 |
| TRIM48 | cg02204046 | 11 | 0.37±0.05 | 0.35±0.06 | 3.85E-02 |
| LDHAL6A | cg02906238 | 11 | 0.67±0.09 | 0.63±0.1 | 2.11E-02 |
| LDHC | cg03270167 | 11 | 0.4±0.1 | 0.35±0.1 | 6.27E-03 |
| NALP6 | cg03584646 | 11 | 0.4±0.09 | 0.35±0.11 | 4.04E-03 |
| KCNQ1DN | cg04872689 | 11 | 0.63±0.11 | 0.58±0.13 | 4.22E-02 |
| KCNQ1DN | cg05443740 | 11 | 0.81±0.06 | 0.78±0.07 | 4.00E-03 |
| POU2AF1 | cg05656364 | 11 | 0.66±0.06 | 0.64±0.08 | 3.68E-02 |
| ALX4 | cg06654134 | 11 | 0.28±0.05 | 0.24±0.06 | 2.41E-06 |
| HNT | cg06816106 | 11 | 0.07±0.02 | 0.08±0.04 | 8.99E-03 |
| WT1 | cg06874144 | 11 | 0.32±0.09 | 0.29±0.09 | 3.70E-02 |
| EIF3S5 | cg07230446 | 11 | 0.61±0.05 | 0.63±0.05 | 4.56E-02 |
| ZNF143 | cg09172980 | 11 | 0.12±0.09 | 0.16±0.12 | 1.88E-02 |
| LDHAL6A | cg10098888 | 11 | 0.64±0.08 | 0.59±0.1 | 1.37E-04 |
| MTNR1B | cg10881306 | 11 | 0.32±0.06 | 0.35±0.08 | 2.61E-02 |
| PC | cg11034784 | 11 | 0.5±0.07 | 0.53±0.08 | 2.88E-02 |
| FLJ23514 | cg11206667 | 11 | 0.25±0.06 | 0.28±0.07 | 8.25E-03 |
| FLJ33790 | cg11388238 | 11 | 0.57±0.09 | 0.54±0.08 | 9.59E-03 |
| NRXN2 | cg11618577 | 11 | 0.11±0.04 | 0.09±0.04 | 1.96E-02 |
| SCN4B | cg11884792 | 11 | 0.27±0.08 | 0.24±0.08 | 3.90E-02 |
| IGF2AS | cg12501949 | 11 | 0.08±0.03 | 0.09±0.04 | 1.38E-02 |
| SERGEF | cg13384498 | 11 | 0.06±0.03 | 0.08±0.04 | 4.64E-02 |
| LRDD | cg15144793 | 11 | 0.17±0.03 | 0.18±0.04 | 2.18E-02 |
| UBE4A | cg15377518 | 11 | 0.42±0.09 | 0.39±0.09 | 4.87E-02 |
| LPXN | cg15566222 | 11 | 0.15±0.06 | 0.18±0.08 | 3.62E-02 |
| OVOL1 | cg15808558 | 11 | 0.18±0.08 | 0.22±0.11 | 1.53E-02 |
| EED | cg15872103 | 11 | 0.1±0.06 | 0.14±0.1 | 9.97E-03 |
| TOLLIP | cg17192247 | 11 | 0.83±0.05 | 0.8±0.07 | 6.55E-03 |
| MRGPRF | cg18096388 | 11 | 0.63±0.06 | 0.6±0.06 | 1.02E-02 |
| GSTP1 | cg19971804 | 11 | 0.28±0.07 | 0.25±0.07 | 4.40E-03 |
| FLJ23514 | cg20415809 | 11 | 0.15±0.04 | 0.17±0.05 | 4.78E-03 |
| KCNC1 | cg22380033 | 11 | 0.27±0.07 | 0.25±0.06 | 1.74E-02 |
| LAG3 | cg24478630 | 12 | 0.47±0.08 | 0.49±0.08 | 3.38E-02 |
| C12orf22 | cg25457027 | 12 | 0.45±0.06 | 0.43±0.06 | 4.08E-02 |
| CSAD | cg26394380 | 12 | 0.28±0.14 | 0.34±0.16 | 1.03E-02 |
| KRT7 | cg02413850 | 12 | 0.12±0.04 | 0.14±0.05 | 2.61E-02 |
| DPPA3 | cg04405541 | 12 | 0.52±0.08 | 0.42±0.08 | 9.21E-13 |
| KCNA1 | cg05451974 | 12 | 0.23±0.08 | 0.19±0.08 | 4.01E-03 |
| NEUROD4 | cg06143901 | 12 | 0.13±0.06 | 0.12±0.06 | 4.74E-02 |
| PIWIL1 | cg15056412 | 12 | 0.64±0.12 | 0.69±0.15 | 2.77E-02 |
| KRTHB6 | cg20526800 | 12 | 0.84±0.04 | 0.86±0.03 | 1.24E-04 |
| HOXC12 | cg24587268 | 12 | 0.15±0.03 | 0.17±0.04 | 4.33E-02 |
| MYF5 | cg26806924 | 12 | 0.63±0.08 | 0.57±0.12 | 1.48E-03 |
| SLC11A2 | cg27270218 | 12 | 0.18±0.09 | 0.22±0.12 | 3.73E-02 |
| DYRK2 | cg04297093 | 12 | 0.28±0.08 | 0.31±0.11 | 3.80E-02 |
| C12orf40 | cg06502510 | 12 | 0.73±0.09 | 0.69±0.11 | 2.05E-02 |
| SYT10 | cg11429658 | 12 | 0.34±0.06 | 0.3±0.06 | 7.20E-05 |
| PRPH | cg12186917 | 12 | 0.2±0.04 | 0.21±0.04 | 4.65E-02 |
| RB1 | cg00556408 | 13 | 0.69±0.06 | 0.71±0.06 | 3.52E-02 |
| RASL11A | cg05266460 | 13 | 0.04±0.03 | 0.05±0.04 | 4.58E-02 |
| LIG4 | cg05670348 | 13 | 0.51±0.14 | 0.46±0.14 | 1.46E-02 |
| NUPL1 | cg06534422 | 13 | 0.22±0.06 | 0.17±0.05 | 8.88E-07 |
| WDFY2 | cg09958090 | 13 | 0.06±0.03 | 0.08±0.04 | 4.20E-02 |
| PABPC3 | cg13788301 | 13 | 0.5±0.08 | 0.46±0.09 | 7.17E-03 |
| MGC9850 | cg16189954 | 13 | 0.09±0.05 | 0.12±0.08 | 8.62E-03 |
| RB1 | cg20120491 | 13 | 0.37±0.07 | 0.4±0.07 | 4.54E-02 |
| AP4S1 | cg24104611 | 14 | 0.27±0.06 | 0.3±0.08 | 3.98E-02 |
| AMN | cg02905245 | 14 | 0.79±0.04 | 0.77±0.05 | 1.28E-02 |
| TSHR | cg02929869 | 14 | 0.32±0.06 | 0.3±0.04 | 1.12E-02 |
| DLK1 | cg03075662 | 14 | 0.26±0.05 | 0.28±0.06 | 5.57E-03 |
| SMOC1 | cg03667091 | 14 | 0.13±0.04 | 0.14±0.05 | 4.94E-02 |
| KIAA1446 | cg04835638 | 14 | 0.3±0.06 | 0.27±0.07 | 9.17E-03 |
| C14orf162 | cg05570980 | 14 | 0.82±0.08 | 0.78±0.11 | 4.37E-02 |
| MEG3 | cg05722552 | 14 | 0.59±0.05 | 0.62±0.07 | 1.32E-02 |
| C14orf126 | cg05781767 | 14 | 0.42±0.05 | 0.44±0.05 | 1.91E-02 |
| C14orf162 | cg08375941 | 14 | 0.62±0.1 | 0.57±0.1 | 6.57E-03 |
| ADSSL1 | cg08611205 | 14 | 0.14±0.05 | 0.11±0.05 | 1.56E-03 |
| C14orf149 | cg10864941 | 14 | 0.04±0.02 | 0.05±0.03 | 3.71E-02 |
| BMP4 | cg11158430 | 14 | 0.54±0.09 | 0.51±0.09 | 3.87E-02 |
| SLC7A8 | cg12454167 | 14 | 0.26±0.08 | 0.23±0.08 | 1.33E-02 |
| HSPA2 | cg12507125 | 14 | 0.24±0.08 | 0.21±0.07 | 1.56E-02 |
| LASS3 | cg13515605 | 15 | 0.62±0.2 | 0.69±0.19 | 1.86E-02 |
| ZNF710 | cg13725272 | 15 | 0.38±0.09 | 0.35±0.1 | 3.89E-02 |
| VPS18 | cg14246617 | 15 | 0.19±0.05 | 0.17±0.06 | 2.07E-02 |
| FLJ20582 | cg15798455 | 15 | 0.57±0.11 | 0.44±0.11 | 9.48E-11 |
| ACTC | cg15877314 | 15 | 0.53±0.07 | 0.5±0.06 | 2.28E-02 |
| SPESP1 | cg18215716 | 15 | 0.75±0.07 | 0.7±0.08 | 1.73E-05 |
| FLJ43276 | cg19740375 | 15 | 0.06±0.04 | 0.14±0.04 | 1.82E-21 |
| HERC2 | cg20074371 | 15 | 0.59±0.04 | 0.61±0.05 | 2.27E-02 |
| SNAPC5 | cg21291896 | 15 | 0.65±0.06 | 0.63±0.07 | 2.15E-02 |
| FLJ43276 | cg22084336 | 15 | 0.07±0.04 | 0.14±0.04 | 3.63E-21 |
| NDUFAF1 | cg22470827 | 15 | 0.57±0.12 | 0.51±0.13 | 1.11E-02 |
| ALDH1A3 | cg22959932 | 15 | 0.62±0.07 | 0.59±0.08 | 1.67E-02 |
| SNRPA1 | cg24832353 | 15 | 0.46±0.07 | 0.48±0.07 | 4.84E-02 |
| DAPK2 | cg25103286 | 15 | 0.56±0.13 | 0.51±0.16 | 4.96E-02 |
| FGF7 | cg25345005 | 15 | 0.53±0.09 | 0.48±0.1 | 2.30E-03 |
| ISLR2 | cg26647600 | 15 | 0.14±0.06 | 0.12±0.05 | 8.40E-03 |
| BNC1 | cg27226214 | 15 | 0.24±0.07 | 0.21±0.05 | 1.12E-02 |
| CHRNB4 | cg27440834 | 15 | 0.25±0.06 | 0.22±0.06 | 1.70E-02 |
| CLCN7 | cg01528948 | 16 | 0.82±0.05 | 0.8±0.05 | 4.02E-02 |
| SPIN1 | cg02741177 | 16 | 0.06±0.05 | 0.08±0.07 | 4.11E-02 |
| LDHD | cg03883519 | 16 | 0.36±0.14 | 0.3±0.12 | 1.28E-03 |
| CDK10 | cg04249586 | 16 | 0.48±0.11 | 0.45±0.11 | 4.85E-02 |
| PRKCB1 | cg05354432 | 16 | 0.06±0.03 | 0.07±0.04 | 4.66E-02 |
| SRRM2 | cg06425515 | 16 | 0.76±0.11 | 0.71±0.14 | 2.06E-02 |
| OR1F1 | cg08377000 | 16 | 0.81±0.07 | 0.78±0.08 | 2.44E-02 |
| MGC52282 | cg10737625 | 16 | 0.69±0.08 | 0.71±0.06 | 2.70E-02 |
| ASCIZ | cg11277230 | 16 | 0.71±0.06 | 0.69±0.08 | 3.39E-02 |
| SMPD3 | cg11465971 | 16 | 0.59±0.09 | 0.62±0.08 | 1.11E-02 |
| CDK10 | cg13436996 | 16 | 0.35±0.09 | 0.32±0.09 | 4.15E-02 |
| FLJ32130 | cg13850625 | 16 | 0.11±0.08 | 0.17±0.12 | 1.28E-03 |
| TMC7 | cg13996731 | 16 | 0.14±0.04 | 0.12±0.04 | 2.41E-02 |
| FAM57B | cg14561282 | 16 | 0.29±0.06 | 0.27±0.06 | 3.04E-02 |
| COX4I1 | cg18059088 | 16 | 0.35±0.07 | 0.33±0.06 | 4.92E-02 |
| MPG | cg18176712 | 16 | 0.36±0.12 | 0.4±0.15 | 4.84E-02 |
| ZNF553 | cg18277508 | 16 | 0.36±0.06 | 0.34±0.07 | 3.66E-02 |
| FOXL1 | cg19843036 | 16 | 0.65±0.08 | 0.61±0.1 | 2.72E-02 |
| C16orf30 | cg20391984 | 16 | 0.6±0.19 | 0.53±0.21 | 2.46E-02 |
| PRSS22 | cg21094669 | 16 | 0.15±0.1 | 0.19±0.12 | 3.34E-02 |
| SMPD3 | cg22064942 | 16 | 0.57±0.11 | 0.54±0.1 | 2.64E-02 |
| PRSS22 | cg22127491 | 16 | 0.05±0.02 | 0.07±0.04 | 8.90E-03 |
| IL21R | cg22182945 | 16 | 0.18±0.09 | 0.21±0.1 | 3.88E-02 |
| RBM35B | cg23207990 | 16 | 0.16±0.05 | 0.14±0.06 | 3.00E-02 |
| ASPHD1 | cg23322316 | 16 | 0.43±0.09 | 0.38±0.07 | 2.99E-04 |
| FAM100A | cg25201363 | 16 | 0.17±0.04 | 0.16±0.04 | 1.98E-02 |
| MT1F | cg26647453 | 16 | 0.09±0.04 | 0.11±0.05 | 4.59E-02 |
| TM4SF11 | cg27558666 | 16 | 0.22±0.06 | 0.2±0.05 | 2.59E-02 |
| SMPD3 | cg27563778 | 16 | 0.15±0.07 | 0.12±0.07 | 1.08E-02 |
| LDHD | cg01058368 | 16 | 0.45±0.12 | 0.4±0.11 | 2.68E-03 |
| DUS2L | cg01278291 | 16 | 0.06±0.02 | 0.07±0.04 | 3.86E-02 |
| FLJ43855 | cg02196805 | 16 | 0.78±0.05 | 0.81±0.05 | 1.79E-03 |
| DEXI | cg03723845 | 16 | 0.37±0.09 | 0.32±0.1 | 3.42E-03 |
| FMNL1 | cg05382565 | 17 | 0.15±0.07 | 0.18±0.08 | 3.07E-02 |
| FOXJ1 | cg06225581 | 17 | 0.12±0.03 | 0.13±0.03 | 3.59E-02 |
| PRAC | cg06501790 | 17 | 0.12±0.04 | 0.14±0.05 | 1.64E-02 |
| CCL8 | cg06609475 | 17 | 0.34±0.09 | 0.31±0.09 | 3.87E-02 |
| ASPA | cg07172256 | 17 | 0.51±0.1 | 0.48±0.1 | 2.93E-02 |
| SMYD4 | cg09262269 | 17 | 0.07±0.06 | 0.1±0.09 | 2.55E-02 |
| HEXIM1 | cg10038259 | 17 | 0.66±0.08 | 0.63±0.08 | 3.23E-02 |
| XYLT2 | cg10660256 | 17 | 0.4±0.09 | 0.36±0.12 | 2.46E-02 |
| ZPBP2 | cg10988561 | 17 | 0.62±0.08 | 0.58±0.08 | 4.15E-04 |
| LOC201164 | cg11151820 | 17 | 0.12±0.08 | 0.17±0.11 | 1.01E-02 |
| CROP | cg11248413 | 17 | 0.07±0.03 | 0.08±0.05 | 3.69E-02 |
| LYK5 | cg13284426 | 17 | 0.19±0.07 | 0.17±0.06 | 4.83E-02 |
| MGAT5B | cg13703437 | 17 | 0.35±0.06 | 0.33±0.07 | 4.79E-02 |
| ZNF207 | cg15091410 | 17 | 0.14±0.07 | 0.17±0.09 | 1.24E-02 |
| PTRH2 | cg15563057 | 17 | 0.07±0.05 | 0.1±0.08 | 1.69E-02 |
| SECTM1 | cg17225169 | 17 | 0.5±0.1 | 0.45±0.13 | 3.43E-02 |
| RAB37 | cg20708411 | 17 | 0.08±0.03 | 0.09±0.04 | 1.40E-02 |
| ARHGAP27 | cg21046940 | 17 | 0.72±0.07 | 0.69±0.08 | 4.40E-02 |
| ZNF232 | cg21410991 | 17 | 0.59±0.13 | 0.54±0.14 | 2.14E-02 |
| THRAP4 | cg24043192 | 17 | 0.53±0.09 | 0.48±0.1 | 1.08E-02 |
| FN3KRP | cg24136586 | 17 | 0.36±0.09 | 0.33±0.1 | 3.75E-02 |
| ZPBP2 | cg24382521 | 17 | 0.77±0.04 | 0.74±0.06 | 7.69E-05 |
| LYZL6 | cg27092035 | 17 | 0.71±0.07 | 0.68±0.08 | 4.81E-02 |
| RAD51C | cg01907837 | 17 | 0.71±0.09 | 0.67±0.1 | 2.62E-02 |
| CSNK1D | cg02486845 | 17 | 0.16±0.04 | 0.14±0.05 | 6.52E-03 |
| SUMO2 | cg02500392 | 17 | 0.44±0.09 | 0.49±0.1 | 3.91E-03 |
| SRP68 | cg04600618 | 17 | 0.62±0.08 | 0.59±0.09 | 1.52E-02 |
| CCDC42 | cg04946916 | 17 | 0.06±0.03 | 0.07±0.05 | 4.83E-02 |
| RAB5C | cg05262335 | 17 | 0.44±0.08 | 0.41±0.09 | 2.62E-02 |
| SLC6A4 | cg05598246 | 17 | 0.32±0.09 | 0.27±0.09 | 1.94E-03 |
| SP6 | cg06334093 | 17 | 0.68±0.07 | 0.71±0.07 | 1.69E-02 |
| CAMKK1 | cg06656924 | 17 | 0.27±0.09 | 0.3±0.12 | 3.99E-02 |
| EVPL | cg07971188 | 17 | 0.28±0.09 | 0.24±0.1 | 4.81E-03 |
| CCDC55 | cg08636224 | 17 | 0.05±0.03 | 0.07±0.05 | 4.03E-02 |
| AATF | cg08946989 | 17 | 0.08±0.05 | 0.11±0.08 | 2.12E-02 |
| CACNG4 | cg09871043 | 17 | 0.38±0.1 | 0.34±0.12 | 3.46E-02 |
| TEKT3 | cg10301967 | 17 | 0.08±0.04 | 0.1±0.06 | 3.53E-02 |
| MPPE1 | cg11787828 | 18 | 0.05±0.03 | 0.06±0.05 | 4.96E-02 |
| RALBP1 | cg15451100 | 18 | 0.22±0.07 | 0.18±0.06 | 1.22E-04 |
| KIAA0427 | cg15926557 | 18 | 0.49±0.07 | 0.47±0.08 | 3.49E-02 |
| C19orf33 | cg19318326 | 19 | 0.39±0.13 | 0.35±0.12 | 3.73E-02 |
| PSG5 | cg20095587 | 19 | 0.73±0.05 | 0.75±0.05 | 2.68E-02 |
| GALP | cg20811607 | 19 | 0.67±0.09 | 0.64±0.1 | 4.33E-02 |
| GPATC1 | cg21269897 | 19 | 0.07±0.03 | 0.09±0.03 | 1.36E-02 |
| FBXO27 | cg21453677 | 19 | 0.25±0.06 | 0.23±0.05 | 1.97E-02 |
| ZNF228 | cg23070249 | 19 | 0.18±0.05 | 0.15±0.04 | 3.00E-05 |
| CRB3 | cg23070585 | 19 | 0.4±0.06 | 0.38±0.06 | 1.55E-02 |
| RAB8A | cg23696834 | 19 | 0.72±0.07 | 0.69±0.08 | 1.06E-02 |
| TNFSF14 | cg25181693 | 19 | 0.68±0.06 | 0.71±0.07 | 2.87E-02 |
| MOBKL2A | cg27573806 | 19 | 0.49±0.08 | 0.52±0.1 | 1.39E-02 |
| CSPG3 | cg00053647 | 19 | 0.6±0.07 | 0.56±0.07 | 4.20E-04 |
| ELA2 | cg00503840 | 19 | 0.15±0.07 | 0.18±0.09 | 3.17E-02 |
| ATP5D | cg00548268 | 19 | 0.55±0.1 | 0.51±0.13 | 2.97E-02 |
| ZIM2 | cg00644033 | 19 | 0.64±0.05 | 0.67±0.06 | 8.22E-03 |
| TIP39 | cg00884529 | 19 | 0.21±0.05 | 0.2±0.05 | 4.20E-02 |
| C19orf33 | cg00967316 | 19 | 0.16±0.09 | 0.13±0.08 | 4.88E-02 |
| P2RY11 | cg01182585 | 19 | 0.2±0.1 | 0.24±0.13 | 4.13E-02 |
| IL27RA | cg01430430 | 19 | 0.11±0.07 | 0.14±0.1 | 3.95E-02 |
| SHKBP1 | cg03148461 | 19 | 0.18±0.05 | 0.2±0.07 | 3.63E-02 |
| CGB2 | cg05660795 | 19 | 0.4±0.07 | 0.42±0.08 | 4.26E-02 |
| CLEC11A | cg08445039 | 19 | 0.22±0.05 | 0.21±0.05 | 3.42E-02 |
| LGALS7 | cg08744726 | 19 | 0.2±0.07 | 0.22±0.07 | 2.73E-02 |
| PEG3 | cg10880863 | 19 | 0.65±0.05 | 0.68±0.06 | 6.35E-03 |
| AXL | cg10971269 | 19 | 0.3±0.08 | 0.27±0.09 | 4.55E-02 |
| ZNF545 | cg11513856 | 19 | 0.64±0.08 | 0.61±0.1 | 4.79E-02 |
| CGB2 | cg12567315 | 19 | 0.66±0.05 | 0.67±0.05 | 3.39E-02 |
| NALP2 | cg12614105 | 19 | 0.71±0.15 | 0.75±0.12 | 4.57E-02 |
| USP29 | cg13289321 | 19 | 0.62±0.09 | 0.59±0.09 | 1.71E-02 |
| ZNF264 | cg13456653 | 19 | 0.62±0.07 | 0.59±0.08 | 1.42E-02 |
| ZNF135 | cg13462129 | 19 | 0.08±0.03 | 0.09±0.03 | 1.09E-02 |
| ZNF677 | cg13464062 | 19 | 0.12±0.06 | 0.15±0.07 | 4.07E-02 |
| C3 | cg14820798 | 19 | 0.71±0.06 | 0.68±0.09 | 2.52E-02 |
| ZIM2 | cg14898779 | 19 | 0.61±0.07 | 0.63±0.07 | 3.00E-02 |
| UHRF1 | cg17628717 | 19 | 0.18±0.06 | 0.21±0.08 | 1.15E-02 |
| KLK8 | cg17731261 | 19 | 0.73±0.07 | 0.76±0.07 | 2.04E-03 |
| PEG3 | cg18015677 | 19 | 0.51±0.05 | 0.52±0.06 | 4.63E-02 |
| CGB1 | cg19548738 | 19 | 0.83±0.03 | 0.85±0.03 | 9.50E-03 |
| CKM | cg19747852 | 19 | 0.39±0.1 | 0.35±0.1 | 1.99E-02 |
| LRFN3 | cg19873785 | 19 | 0.56±0.16 | 0.5±0.17 | 4.93E-02 |
| OSCAR | cg20991801 | 19 | 0.68±0.08 | 0.63±0.08 | 5.03E-04 |
| CEACAM6 | cg21041127 | 19 | 0.09±0.05 | 0.12±0.06 | 1.55E-02 |
| MGC11271 | cg21636748 | 19 | 0.28±0.06 | 0.25±0.05 | 5.74E-03 |
| TOMM40 | cg21784940 | 19 | 0.06±0.02 | 0.07±0.04 | 1.70E-02 |
| ZNF541 | cg22878324 | 19 | 0.22±0.09 | 0.2±0.08 | 3.52E-02 |
| GALP | cg24302095 | 19 | 0.79±0.03 | 0.78±0.04 | 4.08E-02 |
| ZNF228 | cg25300386 | 19 | 0.1±0.05 | 0.08±0.04 | 3.09E-03 |
| FBXO17 | cg25778479 | 19 | 0.66±0.05 | 0.61±0.07 | 9.01E-06 |
| SNAPC2 | cg25990230 | 19 | 0.23±0.07 | 0.21±0.04 | 1.04E-02 |
| UBA52 | cg26180557 | 19 | 0.05±0.02 | 0.06±0.04 | 4.52E-02 |
| DHRS10 | cg01692572 | 19 | 0.28±0.14 | 0.33±0.15 | 4.47E-02 |
| ISOC2 | cg03380773 | 19 | 0.04±0.03 | 0.09±0.11 | 2.31E-03 |
| FLJ13265 | cg03835158 | 19 | 0.84±0.04 | 0.83±0.05 | 3.10E-02 |
| EMILIN3 | cg04681849 | 20 | 0.1±0.02 | 0.11±0.04 | 4.84E-02 |
| PRIC285 | cg09378940 | 20 | 0.75±0.09 | 0.78±0.09 | 3.10E-02 |
| RTEL1 | cg10171125 | 20 | 0.46±0.14 | 0.39±0.13 | 3.11E-03 |
| ADRA1D | cg14070647 | 20 | 0.9±0.04 | 0.88±0.04 | 7.11E-03 |
| SLC4A11 | cg15399561 | 20 | 0.3±0.07 | 0.27±0.07 | 2.11E-02 |
| NNAT | cg16793061 | 20 | 0.72±0.07 | 0.75±0.06 | 2.80E-02 |
| MC3R | cg17162024 | 20 | 0.21±0.05 | 0.23±0.06 | 9.44E-03 |
| TFAP2C | cg18640030 | 20 | 0.11±0.03 | 0.12±0.04 | 4.01E-02 |
| R3HDML | cg20845050 | 20 | 0.11±0.03 | 0.09±0.03 | 1.10E-02 |
| ZGPAT | cg21750887 | 20 | 0.08±0.05 | 0.1±0.06 | 4.89E-02 |
| C20orf102 | cg22610305 | 20 | 0.19±0.05 | 0.21±0.04 | 7.34E-03 |
| MMP9 | cg23566335 | 20 | 0.65±0.05 | 0.62±0.06 | 4.12E-04 |
| CD40 | cg23843812 | 20 | 0.13±0.06 | 0.16±0.09 | 1.60E-02 |
| SULF2 | cg24267801 | 20 | 0.22±0.1 | 0.26±0.14 | 2.60E-02 |
| NNAT | cg24698622 | 20 | 0.59±0.09 | 0.62±0.08 | 3.50E-02 |
| FLJ45832 | cg25201980 | 20 | 0.78±0.05 | 0.75±0.06 | 7.27E-04 |
| C20orf30 | cg25510610 | 20 | 0.17±0.04 | 0.19±0.05 | 3.95E-02 |
| MYBL2 | cg26212924 | 20 | 0.12±0.04 | 0.1±0.03 | 2.68E-03 |
| CD40 | cg00047469 | 20 | 0.09±0.05 | 0.11±0.06 | 1.22E-02 |
| WFDC2 | cg00594118 | 20 | 0.11±0.03 | 0.1±0.03 | 3.66E-02 |
| DEFB123 | cg00842351 | 20 | 0.8±0.07 | 0.75±0.1 | 6.86E-03 |
| GM632 | cg01536400 | 20 | 0.43±0.08 | 0.4±0.08 | 4.02E-02 |
| GNAS | cg01618660 | 20 | 0.67±0.05 | 0.69±0.06 | 1.67E-02 |
| DHX35 | cg02009694 | 20 | 0.12±0.03 | 0.14±0.03 | 4.89E-04 |
| SLC19A1 | cg04725234 | 21 | 0.21±0.06 | 0.24±0.08 | 1.44E-02 |
| CLIC6 | cg05413282 | 21 | 0.19±0.07 | 0.21±0.08 | 2.92E-02 |
| RIPK4 | cg05600174 | 21 | 0.28±0.07 | 0.24±0.09 | 1.70E-02 |
| CRYAA | cg05840553 | 21 | 0.3±0.05 | 0.32±0.05 | 4.61E-02 |
| HMGN1 | cg06484397 | 21 | 0.46±0.06 | 0.49±0.08 | 1.59E-02 |
| C21orf81 | cg08876665 | 21 | 0.21±0.09 | 0.17±0.09 | 5.34E-03 |
| EIF3S6IP | cg10922622 | 22 | 0.17±0.05 | 0.15±0.04 | 7.88E-04 |
| POLR3H | cg11809085 | 22 | 0.66±0.11 | 0.62±0.13 | 2.35E-02 |
| PNPLA5 | cg12044689 | 22 | 0.21±0.04 | 0.2±0.03 | 3.81E-03 |
| SLC5A1 | cg12622986 | 22 | 0.52±0.13 | 0.57±0.13 | 1.29E-02 |
| FBLN1 | cg12927617 | 22 | 0.34±0.09 | 0.3±0.1 | 7.32E-03 |
| PRAME | cg13309018 | 22 | 0.4±0.06 | 0.38±0.06 | 4.87E-02 |
| H1F0 | cg14643978 | 22 | 0.2±0.05 | 0.17±0.05 | 1.30E-04 |
| AIFL | cg14776416 | 22 | 0.19±0.04 | 0.17±0.04 | 1.27E-02 |
| GP1BB | cg14802951 | 22 | 0.22±0.1 | 0.27±0.13 | 8.63E-03 |
| GALR3 | cg22778947 | 22 | 0.85±0.05 | 0.83±0.07 | 4.87E-02 |
| SCO2 | cg24770649 | 22 | 0.12±0.04 | 0.15±0.07 | 2.12E-02 |

Note: red indicates the gene methylated sex differentially in both saliva and peripheral blood cell.
