# Supplementary material for: A Study of the Influence of Sex on Genome Wide Methylation
Source: PLoS One. 2010 Apr 6;5(4):e10028. doi: 10.1371/journal.pone.0010028 (PMC2850313; doi:10.1371/journal.pone.0010028)
Supplement: Table S3 — Correlation results before and after sex effect correction. Significant correlation of methylation with age, marijuana use, maximum drink, and cigarette use before and after sex effect correction. (0.14 MB DOC) [file pone.0010028.s003.doc]

Supporting Table S3: Correlation results before and after sex effect correction

| Gene | Target ID | Chr. | Correlation | P value | Methylation  Mean | Methylation standard deviation |
| --- | --- | --- | --- | --- | --- | --- |
| Age | | | | | | |
| EDARADD | cg09809672 | 1 | -0.54 | 1.32E-16 | 0.44 | 0.07 |
| GLRA1 | cg26692016 | 5 | 0.53 | 1E-15 | 0.3 | 0.05 |
| SELP | cg01459453 | 1 | -0.51 | 1.3E-14 | 0.73 | 0.08 |
| KCNG3 | cg10583382 | 2 | 0.49 | 1.8E-13 | 0.28 | 0.06 |
| LEP | cg24306924 | 7 | 0.49 | 2.04E-13 | 0.34 | 0.07 |
| NPDC1 | cg06855803 | 9 | -0.49 | 4.65E-13 | 0.43 | 0.06 |
| FZD9 | cg03184439 | 7 | 0.46 | 5.87E-12 | 0.31 | 0.09 |
| CHD1L | cg11120551 | 1 | -0.46 | 1.26E-11 | 0.27 | 0.05 |
| RHBDD1 | cg15781794 | 2 | 0.46 | 1.38E-11 | 0.1 | 0.03 |
| KCNC1 | cg22380033 | 11 | 0.45 | 1.86E-11 | 0.26 | 0.06 |
| TSGA14 | cg25665528 | 7 | 0.45 | 2.69E-11 | 0.25 | 0.07 |
| SEC31L2 | cg19123863 | 10 | 0.44 | 7.85E-11 | 0.32 | 0.12 |
| PDE4C | cg15447479 | 19 | 0.44 | 9.77E-11 | 0.23 | 0.06 |
| SCAP | cg07215749 | 3 | -0.44 | 1.12E-10 | 0.56 | 0.07 |
| DIRAS3 | cg22901840 | 1 | 0.43 | 1.99E-10 | 0.65 | 0.07 |
| WNK2 | cg03774732 | 9 | 0.43 | 2.53E-10 | 0.11 | 0.05 |
| RTP1 | cg18338296 | 3 | 0.43 | 4.31E-10 | 0.71 | 0.06 |
| RHD | cg24857721 | 1 | -0.41 | 1.55E-09 | 0.69 | 0.11 |
| SOCS1 | cg11768886 | 16 | 0.41 | 1.73E-09 | 0.51 | 0.15 |
| NPTX2 | cg24333473 | 7 | 0.41 | 2.11E-09 | 0.11 | 0.05 |
| PNMT | cg19905251 | 17 | -0.41 | 2.34E-09 | 0.15 | 0.03 |
| HDAC10 | cg13226172 | 22 | -0.41 | 2.85E-09 | 0.21 | 0.06 |
| GREM1 | cg24130043 | 15 | 0.41 | 3.28E-09 | 0.13 | 0.04 |
| PGLYRP2 | cg00677811 | 19 | -0.4 | 4.42E-09 | 0.31 | 0.06 |
| ZNF167 | cg16332060 | 3 | 0.4 | 5.37E-09 | 0.68 | 0.11 |
| C10orf82 | cg11801011 | 10 | 0.4 | 6.49E-09 | 0.15 | 0.04 |
| CCRK | cg01545242 | 9 | 0.39 | 1.2E-08 | 0.22 | 0.06 |
| TFAP2E | cg26372517 | 1 | 0.38 | 2.53E-08 | 0.18 | 0.09 |
| RUNX2 | cg23886551 | 6 | -0.38 | 4.5E-08 | 0.49 | 0.1 |
| NPTX2 | cg11297236 | 7 | 0.38 | 4.77E-08 | 0.32 | 0.06 |
| SCRT1 | cg15979932 | 8 | 0.38 | 4.89E-08 | 0.22 | 0.05 |
| TRIM58 | cg07533148 | 1 | 0.37 | 6.54E-08 | 0.09 | 0.05 |
| TDRD5 | cg09656934 | 1 | 0.37 | 7.16E-08 | 0.31 | 0.08 |
| GATA4 | cg16020904 | 8 | 0.37 | 7.48E-08 | 0.18 | 0.05 |
| FBLN2 | cg10848367 | 3 | 0.37 | 8.42E-08 | 0.17 | 0.06 |
| WT1 | cg26897054 | 11 | 0.37 | 8.65E-08 | 0.24 | 0.08 |
| SLC10A4 | cg13628514 | 4 | 0.37 | 9.84E-08 | 0.15 | 0.03 |
| SOX18 | cg01549015 | 20 | -0.37 | 1.07E-07 | 0.58 | 0.12 |
| RGS14 | cg27537561 | 5 | -0.37 | 1.08E-07 | 0.11 | 0.04 |
| HKR1 | cg06745740 | 19 | 0.36 | 1.66E-07 | 0.31 | 0.14 |
| PKMYT1 | cg02782630 | 16 | 0.36 | 1.91E-07 | 0.45 | 0.07 |
| PLEKHF1 | cg25887294 | 19 | -0.36 | 1.94E-07 | 0.27 | 0.08 |
| GBP1 | cg13406950 | 1 | -0.36 | 2.52E-07 | 0.23 | 0.07 |
| ATP8A2 | cg09954385 | 13 | 0.36 | 2.68E-07 | 0.22 | 0.04 |
| DIRAS3 | cg13697378 | 1 | 0.36 | 2.9E-07 | 0.66 | 0.06 |
| TBX6 | cg16418329 | 16 | -0.35 | 3.11E-07 | 0.16 | 0.07 |
| NEFH | cg11959435 | 22 | 0.35 | 3.81E-07 | 0.22 | 0.05 |
| GATA4 | cg24124977 | 8 | 0.35 | 3.94E-07 | 0.53 | 0.09 |
| RAB4A | cg06646021 | 1 | 0.35 | 4.56E-07 | 0.7 | 0.09 |
| HTR7 | cg11158440 | 10 | 0.35 | 5.24E-07 | 0.11 | 0.04 |
| RP11-49G10.8 | cg06093279 | 20 | 0.35 | 5.55E-07 | 0.4 | 0.09 |
| NMBR | cg07478122 | 6 | 0.35 | 5.6E-07 | 0.18 | 0.04 |
| SH3TC1 | cg13338132 | 4 | -0.35 | 6.1E-07 | 0.38 | 0.12 |
| ITGA2B | cg09106817 | 17 | -0.35 | 6.87E-07 | 0.69 | 0.09 |
| CYGB | cg04099420 | 17 | -0.34 | 7.6E-07 | 0.34 | 0.12 |
| GAS2L2 | cg16983159 | 17 | -0.34 | 7.87E-07 | 0.34 | 0.07 |
| RPL39L | cg17259265 | 3 | 0.34 | 7.92E-07 | 0.52 | 0.11 |
| NOC2L | cg19923810 | 1 | 0.34 | 8.07E-07 | 0.79 | 0.07 |
| RPL31 | cg05676042 | 2 | 0.34 | 8.39E-07 | 0.5 | 0.09 |
| ELOVL1 | cg16858125 | 1 | 0.34 | 8.62E-07 | 0.54 | 0.1 |
| DIRAS3 | cg09118625 | 1 | 0.34 | 9.28E-07 | 0.74 | 0.09 |
| TRPV4 | cg14858551 | 12 | 0.34 | 9.36E-07 | 0.36 | 0.07 |
| XPNPEP2 | cg22010317 | X | 0.34 | 9.68E-07 | 0.46 | 0.13 |
| CBFA2T3 | cg15401952 | 16 | -0.34 | 9.95E-07 | 0.43 | 0.14 |
| AKT3 | cg11314684 | 1 | -0.34 | 1.01E-06 | 0.3 | 0.06 |
| SP2 | cg23863670 | 17 | 0.34 | 1.02E-06 | 0.47 | 0.09 |
| CX40.1 | cg07494047 | 10 | -0.34 | 1.29E-06 | 0.58 | 0.06 |
| TRIM50C | cg14696348 | 7 | 0.34 | 1.3E-06 | 0.54 | 0.17 |
| C17orf76 | cg07715201 | 17 | 0.34 | 1.33E-06 | 0.65 | 0.08 |
| METTL7A | cg22954265 | 12 | -0.34 | 1.35E-06 | 0.18 | 0.06 |
| ACTC | cg15877314 | 15 | 0.34 | 1.46E-06 | 0.51 | 0.06 |
| FLJ32065 | cg02944057 | 17 | -0.33 | 1.51E-06 | 0.09 | 0.03 |
| B3GALT6 | cg19945840 | 1 | 0.33 | 1.56E-06 | 0.45 | 0.05 |
| SLC22A18 | cg14290291 | 11 | 0.33 | 1.64E-06 | 0.85 | 0.05 |
| UCN | cg03116740 | 2 | 0.33 | 1.72E-06 | 0.52 | 0.12 |
| PTPN18 | cg09061733 | 2 | -0.33 | 1.73E-06 | 0.16 | 0.04 |
| OTOS | cg13800022 | 2 | 0.33 | 1.9E-06 | 0.75 | 0.05 |
| LAG3 | cg24478630 | 12 | -0.33 | 1.91E-06 | 0.49 | 0.08 |
| ACVR1 | cg27315279 | 2 | -0.33 | 1.97E-06 | 0.4 | 0.09 |
| HTR3E | cg26202340 | 3 | 0.33 | 2.06E-06 | 0.33 | 0.07 |
| FLJ46365 | cg23894539 | 8 | -0.33 | 2.09E-06 | 0.45 | 0.1 |
| SEMA3B | cg20737712 | 3 | -0.33 | 2.14E-06 | 0.18 | 0.08 |
| SHANK2 | cg05218490 | 11 | 0.33 | 2.19E-06 | 0.41 | 0.13 |
| HIST1H1A | cg19287277 | 6 | 0.33 | 2.23E-06 | 0.53 | 0.12 |
| PTGER1 | cg04992673 | 19 | 0.33 | 2.3E-06 | 0.67 | 0.07 |
| DIRAS3 | cg21808053 | 1 | 0.33 | 1.56E-06 | 0.49 | 0.07 |
| M.J. use | | | | | | |
| Gene | Target ID | Chr. | Correlation | P value | Methylation  mean | Methylation  SD |
| ALOXE3 | cg04380513 | 17 | 0.35 | 3.78E-07 | 0.05 | 0.03 |
| GNS | cg00695416 | 12 | 0.35 | 6.6E-07 | 0.03 | 0.03 |
| THBS1 | cg23013392 | 15 | 0.34 | 7.37E-07 | 0.1 | 0.05 |
| WNT3 | cg14700821 | 17 | 0.34 | 7.95E-07 | 0.05 | 0.04 |
| RPRM | cg10501065 | 2 | 0.34 | 1.01E-06 | 0.05 | 0.05 |
| KIAA0513 | cg08493463 | 16 | 0.34 | 1.17E-06 | 0.05 | 0.02 |
| MAF1 | cg21491028 | 8 | 0.34 | 1.47E-06 | 0.06 | 0.04 |
| RAD54L | cg17079378 | 1 | 0.33 | 1.75E-06 | 0.02 | 0.02 |
| TCEAL8 | cg19572242 | X | 0.36 | 2.54E-07 | 0.23 | 0.03 |
| TIMM8A | cg17542495 | X | 0.35 | 4.17E-07 | 0.18 | 0.04 |
| NOV | cg13504059 | 8 | 0.33 | 2.21E-06 | 0.13 | 0.04 |
| Max_drink | | | | | | |
| Gene | Target ID | Chr. | Correlation | P value | Methylation  Mean | Methylation  SD |
| PAGE4 | cg13815872 | X | -0.36 | 1.5E-07 | 0.91 | 0.07 |
| GRM6 | cg03021690 | 5 | 0.34 | 7.3E-07 | 0.07 | 0.03 |
| Cigarette use | | | | | | |
| Gene | Target ID | Chr. | Correlation | P value | Methylation  mean | Methylation  SD |
| SEC31L2 | cg19123863 | 10 | 0.34 | 1.2E-06 | 0.32 | 0.12 |

* The differences between before and after correction are highlighted; Red indicates sites unselected after correction; yellow indicates sites added after correction.
